# Repetitive transcranial magnetic stimulation for bipolar depression: a systematic review and pairwise and network meta-analysis

**DOI:** 10.1038/s41380-023-02045-8

**Published:** 2023-04-05

**Authors:** Taro Kishi, Toshikazu Ikuta, Kenji Sakuma, Masakazu Hatano, Yuki Matsuda, Shinsuke Kito, Nakao Iwata

**Affiliations:** 1 Department of Psychiatry, Fujita Health University School of Medicine, Toyoake, Aichi 470-1192 Japan; 2 Department of Communication Sciences and Disorders, School of Applied Sciences, University of Mississippi, University, Oxford, MS 38677 USA; 3 Department of Clinical Pharmacy, Fujita Health University School of Medicine, Toyoake, Aichi 470–1192 Japan; 4 Department of Psychiatry, Jikei University School of Medicine, Minato-ku, Tokyo 105-8461 Japan; 5 Department of Psychiatry, National Center of Neurology and Psychiatry, 4-1-1 Ogawa-Higashi, Kodaira, Tokyo 187-8551 Japan

**Keywords:** Bipolar disorder, Psychiatric disorders

## To the Editor:

Recent pairwise meta-analysis (PMA) demonstrated that repetitive transcranial magnetic stimulation (rTMS) was effective in treating individuals with major depressive disorder but not bipolar depression (BDep) [1]. However, as only four randomized sham-controlled trials (RSCTs) for BDep were documented, the results may not be robust. Currently, 12 RSCTs of rTMS for treating individuals with BDep have been published (Table S1). Therefore, we conducted a comprehensive systematic review and a random-effects model [2] PMA of rTMS for efficacy, acceptability, and safety outcomes according to the Preferred Reporting Items for Systematic Reviews and Meta-Analyses (PRISMA) statement (Table S2.1) [3]. We also registered it with the Open Science Framework (https://osf.io/t78rv).

Our PMA included RSCTs conducted in adults with BDep. The outcomes were treatment response (primary), improvement in depressive symptoms, remission rate, all-cause discontinuation, and mania incidence. We calculated the risk ratio (RR) for dichotomous variables and the standardized mean difference (SMD) for continuous variables with 95% confidence intervals (95% CI). We also assessed the heterogeneity of the studies using the *I*^2^ statistic, with an *I*^2^ of ≥50% indicating heterogeneity [4]. When the PMA showed significant differences in the treatment response between the treatment groups, the number needed to treat tobenefit (NNTB) was estimated. We used Review Manager software (version 5.4 for Windows; Cochrane Collaboration, http://tech.cochrane.org/Revman) for statistical analyses.

**Fig. 1:**
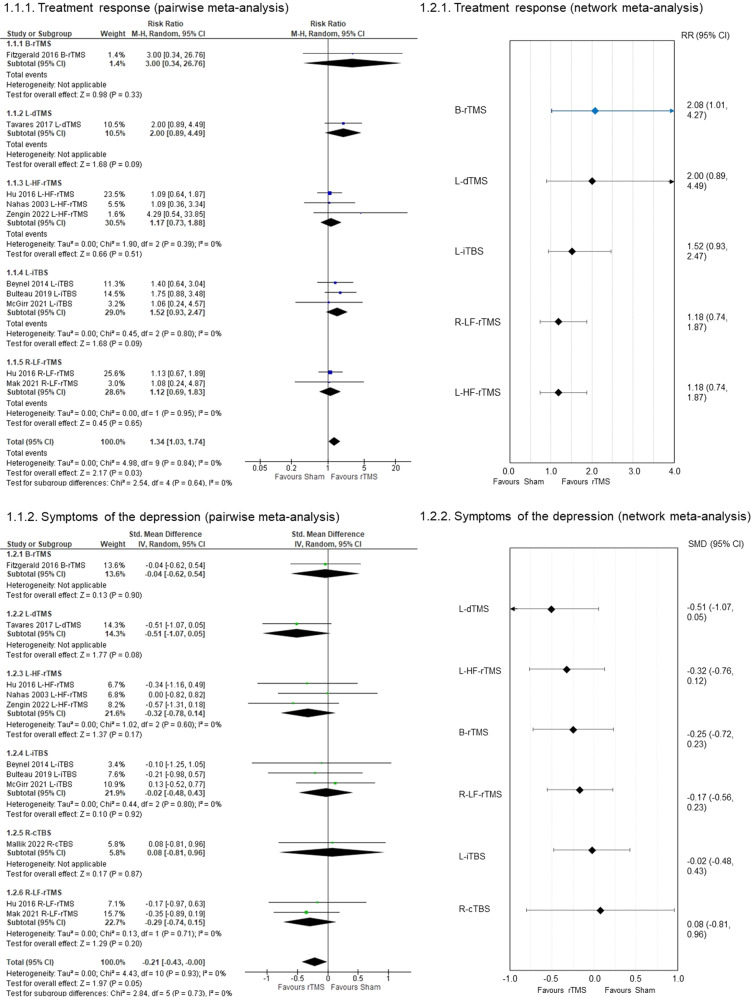
Forest plots. **1.1.1** Treatment response (pairwise meta-analysis). **1.1.2** Symptoms of the depression (pairwise meta-analysis). 95% CI 95% confidence interval, B-rTMS: bilateral repetitive transcranial magnetic stimulation, L-dTMS: left-deep transcranial magnetic stimulation, L-HF-rTMS: left-high frequency-repetitive transcranial magnetic stimulation, L-iTBS: left-intermittent theta burst stimulation, R-cTBS: right-continuous intermittent theta burst stimulation, R-LF-rTMS: right-low frequency-repetitive transcranial magnetic stimulation. **1.2.1** Treatment response (network meta-analysis). **1.2.2** Symptoms of depression (network meta-analysis). The rTMSs were compared with the sham. Colors indicate the presence or absence of a significant difference: blue, the rTMS was superior to the sham; black, the rTMS was similar to the sham. Treatments were ranked according to their surface under the curve cumulative ranking probabilities. 95% CI 95% confidence interval, B-rTMS bilateral repetitive transcranial magnetic stimulation, L-dTMS left-deep transcranial magnetic stimulation, L-HF-rTMS left-high frequency-repetitive transcranial magnetic stimulation, L-iTBS left-intermittent theta burst stimulation, R-cTBS right-continuous intermittent theta burst stimulation, R-LF-rTMS right-low frequency-repetitive transcranial magnetic stimulation, RR risk ratio, SMD standardized mean difference.

Fig. S1 shows the literature search and selection strategy. Tables S1 and S3 summarizes the characteristics of the 12 RSCTs. The rTMS group received bilateral rTMS (B-rTMS, *K* = 1), left-deep TMS (L-dTMS, *K* = 1), left-high frequency-rTMS (*K* = 3), left-intermittent theta burst stimulation (L-iTBS, *K* = 3), right-continuous theta burst stimulation (*K* = 1), right-low frequency-rTMS (R-LF-rTMS, *K* = 3), and rTMS with unknown details (*K* = 1, the study was excluded for analysis). No studies have a high risk of bias in the analysis using the Risk of Bias 2 tool (Fig. S2). Pooled rTMS treatments outperformed a sham to treatment response (RR = 1.34, 95% CI: 1.03, 1.74, *p* = 0.03, *I*^2^ = 0%, NNTB = 8, Fig. 1), and improvement in symptoms of depression (SMD = −0.21, 95% CI: − 0.43, –0.00, *p* = 0.05, *I*^2^ = 0%, Fig. 1). Moreover, L-dTMS and L-iTBS were marginally superior to a sham on treatment response (Fig. 1). L-dTMS was also marginally superior to a sham for improvement in depression (Fig. 1). The pooled rTMS and individual rTMS treatments did not outperform a sham to other outcomes (Appendix S3–S5). Besides, we did not detect a significant publication bias for the primary outcome.

To identify the better rTMS treatments for efficacy, acceptability, and safety of adult individuals with BDep, we then performed a frequentist network meta-analysis (NMA) [5] that allows us to compare three or more interventions simultaneously in a single analysis by combining both direct and indirect evidence across a network of studies [4]. A NMA also produces estimates of the relative effects between any pair of interventions in the network and yields more precise estimates than a single direct or indirect estimate, thereby allowing the estimation of the ranking and hierarchy of interventions [4]. Our NMA conducted based on the PRISMA statement for a NMA (Table S2.2) [6] and used a random-effects model [2]. We also registered it with the Open Science Framework (https://osf.io/sjmhw). We used a similar PICO strategy for NMA and pairwise meta-analysis. However, our NMA included the RSCTs that were also used in our PMA and one head-to-head randomized trial (Fig. S1) [7]. The effect size measures were RR and SMD with 95% CI. Network heterogeneity was assessed using *τ*^2^ statistics. Statistical evaluation of incoherence was performed using the design-by-treatment test (globally) [4] and the Separate Direct from Indirect Evidence test (locally) [4]. The transitivity assumption was tested by extracting potential effect modifiers and comparing their distribution across comparisons in the network. We also performed a meta-regression analysis to detect the association between potential modifiers and the effect size of the primary outcome. Finally, the findings were incorporated into the Confidence in Network Meta-Analysis (CINeMA) application, an adaptation of the Grading of Recommendations Assessment, Development, and Evaluation approach, to assess the credibility of the findings of each of the NMAs [8].

B-rTMS outperformed a sham on treatment response (RR = 2.08, 95% CI: 1.01, 4.27), although the result had considerable heterogeneity. The magnitude of effect sizes of PMA and NMA for L-dTMS and L-iTBS that might be effective for BDep in PMA was similar. Moreover, no potentially confounding factor associated with the primary outcome was observed in meta-regression analyses (Appendix S1). Heterogeneity was not reduced despite adjustments for any potentially confounding factors in a meta-regression (Appendix S1). Thus, no clear evidence of violations of the transitivity assumption for any potential effect modifiers was observed (Table S4 and Appendix S1). Moreover, no significant differences were observed in other outcomes among the treatments (Appendix S2–S5). For all outcomes, global heterogeneity was low, and the network did not show significant global inconsistency. Moreover, there were no statistical agreements in all outcomes between direct and indirect estimates. However, the within-study bias of most of the comparisons was evaluated as “Some concerns.” Moreover, all comparisons for publication bias were evaluated as “Suspected” because funnel plots with fewer than 10 studies were not meaningful [4]. Consequently, confidence in the evidence was generally evaluated as low or very low.

Our PMAs demonstrated novel evidence that rTMS is effective for BDep. Additionally, NMA revealed that B-rTMS had efficacy for individuals with BDep, although the result had considerable heterogeneity. An efficacy trend was also observed in individuals with BDep treated with L-dTMS and L-iTBS. However, the findings of NMA were not conclusive because of the small sample size of the trial. Therefore, a replication randomized trial of B-rTMS, L-dTMS, and L-iTBS should be conducted using a larger sample size. Our study has some limitations. First, our results might include a small-study effect. Moreover, the medications of the individuals included in our meta-analysis differed (Table S1). Finally, the efficacy and safety of accelerated iTBS for BDep, which has been reported to be strongly effective for depression [9], needs to be verified.

### Supplementary information

Supplementary material

## Data Availability

The data used for the current study were reported in the articles of the studies included in our meta-analysis.
