# Supplementary material for: Repetitive transcranial magnetic stimulation for bipolar depression: a systematic review and pairwise and network meta-analysis
Source: Mol Psychiatry. 2023 Apr 5;29(1):39–42. doi: 10.1038/s41380-023-02045-8 (PMC11078724; doi:10.1038/s41380-023-02045-8)
Supplement: Supplementary file 1 — Supplementary material [file 41380_2023_2045_MOESM1_ESM.pdf]

Figure S1: Preferred Reporting Items for Systematic reviews and Meta-Analyses flow diagram

We searched the PubMed, the Cochrane Library, and Embase databases for studies published before February 11, 2023, without language restriction. The search terms for PubMed and the Cochrane Library included (theta burst OR theta-burst OR TBS OR iTBS OR TMS OR rTMS OR repetitive transcranial magnetic stimulation OR transcranial magnetic stimulation) AND (bipolar) AND (random*). The search terms for Embase included ('bipolar disorder'/exp OR 'bipolar disorder') AND ('repetitive transcranial magnetic stimulation'/exp OR 'repetitive transcranial magnetic stimulation' OR 'theta burst stimulation'/exp OR 'theta burst stimulation') AND ('randomized controlled trial'/exp OR 'randomized controlled trial'). Additionally, reference lists of the included articles were manually searched for additional relevant published and unpublished research, including conference abstracts. We also searched clinical trial registries (ClinicalTrials.gov [http://clinicaltrials.gov/] and the World Health Organization International Clinical Trials Registry Platform [http://www.who.int/ictrp/search/en/]) to ensure the RCTs were comprehensive and to minimize the effect of publication bias. Any discrepancies in the selected articles were resolved by consensus of the authors. If multiple papers or academic conference abstracts were reported despite the same research, the literature was screened by confirming the clinical trial registration number and/or reference to past review articles. Of the 336 articles initially identified, 93 were duplicates, 225 were excluded after reviewing the titles and abstracts, and 5 were excluded after reviewing the full texts (two post hoc studies and three studies included individuals with BD and other disorders). In total, 13 articles on eligible studies were selected, and no article was detected from previous review articles. Of 13 eligible RCTs, the Tamas study did not provide the data available for performing a meta-analysis. As the Kazemi study was a head-to-head trial (bilateral-rTMS vs. right-high frequency-rTMS), the RCT was included in a network meta-analysis but not in a pairwise meta-analysis.

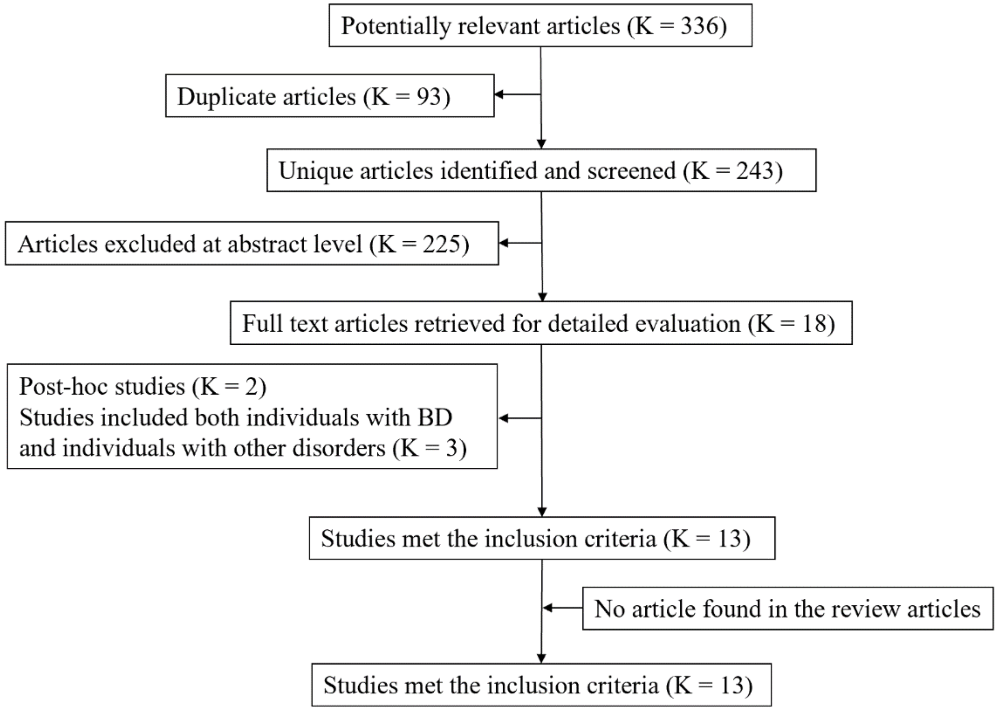

<u>Review articles that we read for the literature search</u>

1. Hyde J, Carr H, Kelley N, Seneviratne R, Reed C, Parlatini V et al. Efficacy of neurostimulation across mental disorders: systematic review and meta-analysis of 208 randomized controlled trials. Mol Psychiatry 2022; 27(6): 2709-2719.

2. Konstantinou G, Hui J, Ortiz A, Kaster TS, Downar J, Blumberger DM et al. Repetitive transcranial magnetic stimulation (rTMS) in bipolar disorder: A systematic review. Bipolar Disord 2022; 24(1): 10-26.

3. Li H, Cui L, Li J, Liu Y, Chen Y. Comparative efficacy and acceptability of neuromodulation procedures in the treatment of treatment-resistant depression: a network meta-analysis of randomized controlled trials. J Affect Disord 2021; 287: 115-124.

4. McGirr A, Karmani S, Arsappa R, Berlim MT, Thirthalli J, Muralidharan K et al. Clinical efficacy and safety of repetitive transcranial magnetic stimulation in acute bipolar depression. World Psychiatry 2016; 15(1): 85-86.

5. Mutz J. Brain stimulation treatment for bipolar disorder. Bipolar Disord 2022.

6. Mutz J, Edgcumbe DR, Brunoni AR, Fu CHY. Efficacy and acceptability of non-invasive brain stimulation for the treatment of adult unipolar and bipolar depression: A systematic review and meta-analysis of randomised sham-controlled trials. Neurosci Biobehav Rev 2018; 92: 291-303.

7. Mutz J, Vipulananthan V, Carter B, Hurlemann R, Fu CHY, Young AH. Comparative efficacy and acceptability of non-surgical brain stimulation for the acute treatment of major depressive episodes in adults: systematic review and network meta-analysis. BMJ 2019; 364: l1079.

8. Nguyen TD, Hieronymus F, Lorentzen R, McGirr A, Ostergaard SD. The efficacy of repetitive transcranial magnetic stimulation (rTMS) for bipolar depression: A systematic review and meta-analysis. J Affect Disord 2021; 279: 250-255.

9. Rosson S, de Filippis R, Croatto G, Collantoni E, Pallottino S, Guinart D et al. Brain stimulation and other biological non-pharmacological interventions in mental disorders: An umbrella review. Neurosci Biobehav Rev 2022; 139: 104743.

<u>Articles included in our systematic review</u>

1. Beynel L, Chauvin A, Guyader N, Harquel S, Szekely D, Bougerol T et al. What saccadic eye movements tell us about TMS-induced neuromodulation of the DLPFC and mood changes: a pilot study in bipolar disorders. Front Integr Neurosci 2014; 8: 65.

2. Bulteau S, Beynel L, Marendaz C, Dall'Igna G, Pere M, Harquel S et al. Twice-daily neuronavigated intermittent theta burst stimulation for bipolar depression: A Randomized Sham-Controlled Pilot Study. Neurophysiol Clin 2019; 49(5): 371-375.

3. Dolberg OT, Dannon PN, Schreiber S, Grunhaus L. Transcranial magnetic stimulation in patients with bipolar depression: a double blind, controlled study. Bipolar Disord 2002; 4 Suppl 1: 94-95.

4. Fitzgerald PB, Hoy KE, Elliot D, McQueen S, Wambeek LE, Daskalakis ZJ. A negative double-blind controlled trial of sequential bilateral rTMS in the treatment of bipolar depression. J Affect Disord 2016; 198: 158-162.

5. Hu SH, Lai JB, Xu DR, Qi HL, Peterson BS, Bao AM et al. Efficacy of repetitive transcranial magnetic stimulation with quetiapine in treating bipolar II depression: a randomized, double-blinded, control study. Sci Rep 2016; 6: 30537.

6. Kazemi R, Rostami R, Khomami S, Horacek J, Brunovsky M, Novak T et al. Electrophysiological correlates of bilateral and unilateral repetitive transcranial magnetic stimulation in patients with bipolar depression. Psychiatry Res 2016; 240: 364-375.

7. Mak ADP, Neggers SFW, Leung ONW, Chu WCW, Ho JYM, Chou IWY et al. Antidepressant efficacy of low-frequency repetitive transcranial magnetic stimulation in antidepressant-nonresponding bipolar depression: a single-blind randomized sham-controlled trial. Int J Bipolar Disord 2021; 9(1): 40.

8. Mallik G, Mishra P, Garg S, Dhyani M, Tikka SK, Tyagi P. Safety and Efficacy of Continuous Theta Burst "Intensive" Stimulation in Acute-Phase Bipolar Depression: A Pilot, Exploratory Study. J ECT 2022.

9. McGirr A, Vila-Rodriguez F, Cole J, Torres IJ, Arumugham SS, Keramatian K et al. Efficacy of Active vs Sham Intermittent Theta Burst Transcranial Magnetic Stimulation for Patients With Bipolar Depression: A Randomized Clinical Trial. JAMA Netw Open 2021; 4(3): e210963.

10. Nahas Z, Kozel FA, Li X, Anderson B, George MS. Left prefrontal transcranial magnetic stimulation (TMS) treatment of depression in bipolar affective disorder: a pilot study of acute safety and efficacy. Bipolar Disord 2003; 5(1): 40-47.

11. Tamas RL, Menkes D, El-Mallakh RS. Stimulating research: a prospective, randomized, double-blind, sham-controlled study of slow transcranial magnetic stimulation in depressed bipolar patients. J Neuropsychiatry Clin Neurosci 2007; 19(2): 198-199.

12. Tavares DF, Myczkowski ML, Alberto RL, Valiengo L, Rios RM, Gordon P et al. Treatment of Bipolar Depression with Deep TMS: Results from a Double-Blind, Randomized, Parallel Group, Sham-Controlled Clinical Trial. Neuropsychopharmacology 2017; 42(13): 2593-2601.

13. Zengin G, Topak OZ, Atesci O, Culha Atesci F. The Efficacy and Safety of Transcranial Magnetic Stimulation in Treatment-Resistant Bipolar Depression. Psychiatr Danub 2022; 34(2): 236-244.

Figure S2: Risk of bias summary

	Randomization process	Deviation from intended intervention	Missing outcome data	Measurement of the outcome	Selection of the reported result	Overall risk of bias
Beynel 2014	Low risk	Low risk	Low risk	Low risk	Some concerns	Some concerns
Bulteau 2019	Some concerns	Low risk	Low risk	Low risk	Some concerns	Some concerns
Dolberg 2002	Some concerns	Low risk	Some concerns	Low risk	Some concerns	Some concerns
Fitzgerald 2016	Low risk	Low risk	Low risk	Low risk	Low risk	Low risk
Hu 2016	Low risk	Low risk	Low risk	Low risk	Low risk	Low risk
Kazemi 2016	Low risk	Some concerns	Low risk	Low risk	Low risk	Some concerns
Mak 2021	Low risk	Some concerns	Low risk	Low risk	Low risk	Some concerns
Mallik 2022	Low risk	Low risk	Low risk	Low risk	Low risk	Low risk
McGirr 2021	Low risk	Low risk	Low risk	Low risk	Low risk	Low risk
Nahas 2003	Some concerns	Some concerns	Low risk	Low risk	Low risk	Some concerns
Tamas 2007	Some concerns	Low risk	Some concerns	Some concerns	Some concerns	Some concerns
Tavares 2017	Low risk	Low risk	Low risk	Low risk	Low risk	Low risk
Zengin 2022	Some concerns	Low risk	Low risk	Low risk	Low risk	Some concerns

Version 2 of the Cochrane risk-of-bias tool for randomized trials (https://www.riskofbias.info/)

Table S1. Characteristics of the randomized, controlled trials in our systematic review and meta-analysis

Study	Sponsorship	Study duration	Diagnosis (BD type)	Minimum of DS at BL	Total n	DS score at BL (mean±SD)	%Female	Age (mean±SD)	Active treatment(s)	Control	Coil	Device	Target	Locating	Frequency (Hz)	MT (%)	Total pulses/session	Total session	Concomitant drug
Beynel 2014	Academia	1~3 w	DSM-IV-TR (I, II, or III)	MADRS>19 [†]	12	MADRS: 30.83±5.46	50.00	51.6±11.7	L-iTBS (twice/d)	Sham coil	Figure-of-eight coil	MagPro	L-DLPFC	MRI	50	80	990	10~30	CYA, HYD, MS,
Bulteau 2019	Academia	3 w	DSM-IV-TR (NR)	MADRS>20	26	MADRS: 29.03±5.61	42.31	52.92± 11.52	L-iTBS (twice/d)	Sham coil	Figure-of-eight coil	MagPro	L-DLPFC	MRI	50	80	990	30	MS (100.00)
Dolberg 2002*	Academia	2 w	NR (NR)**	HAMD>14[†]	20	HAMD: 23.75±6.39	40.00	55.40 ±9.32	rTMS	Sham[‡]	NR	NR	NR	NR	NR	NR	NR	10	NR
Fitzgerald 2016	Industry	4 w	DSM-IV (I or II [56.52%])	HAMD17>2 0	49	HAMD17: 23.10±4.53	56.52	48.00± 11.82	B-rTMS	Sham[§]	Figure-of-eight coil	MagPro	L- and R-DLPFC	MRI	10 (L-DLPFC) and 1 (R-DLPFC)	110	1000 (L-DLPFC) and 1000 (R-DLPFC	20	AD (69.57), AP (58.70), BE (39.13), MS (60.87)
Hu 2016	Academia	4 w	DSM-IV (II [100%])**	HAMD17>2 0	38	HAMD17: 30.00	51.43	26.44± 11.39	(1) L-HF-rTMS and (2) R-LF-rTMS	Sham[§]	Figure-of-eight coil	Magstim	(1) L-DLPFC, (2) R-DLPFC	5.5 cm rule	(1) 10 (L-DLPFC) or 1 (R-DLPFC), (2) 1 (R-DLPFC)	80	1200	20	QUE (100)
Kazemi 2016[†][†]	Academia	6 d	DSM-IV-TR (NR)**	BDI>14	30	BDI: 30.90±8.42	56.67	35.40 ±10.84	(1) B-rTMS and (2) R-LF-rTMS	Non sham	Figure-of-eight coil	Magstim	(1) L- and R-DLPFC, (2) R-DLPFC	5 cm rule	(1) 10 (L-DLPFC) and 1 (R-DLPFC), (2) 1 (R-DLPFC)	120	(1) 3750 (L-DLPFC) and 1500 (R-DLPFC, (2) 2500	20	MS (83.33), SGA (53.33), SSRI (23.33), TRI (80.00)
Mak 2021	Academia	3 w	DSM-5 (I or II [85.19%])	HAMD17>1 9	54	MADRS: 27.24±5.11	66.67	40.03 ±11.26	R-LF-rTMS	Sham coil	Figure-of-eight coil	Magstim	R-DLPFC	MRI	1 (R-DLPFC)	110	300	15	AD (70.37), AP (81.48)
Mallik 2022	Academia	1 w	ICD-10 (NR)**	HAMD>18	19	HAMD17: 27.05±8.87	47.37	39.48 ±12.43	R-cTBS (3 times/d)	Sham coil	Figure-of-eight coil	MagPro	R-DLPFC	10- to 20-EEG	50	80	600	15	AD (10.53), AD+MS (42.11), MS (26.32)
McGirr 2021	Academia	4 w	DSM-5 (I or II [43.24%)	HAMD17>1 7	37	MADRS: 32.97±4.19	62.16	43.86 ±13.87	L-iTBS	Sham coil	Figure-of-eight coil	MagPro	L-DLPFC	MRI or BeamF3	50	120	600	20	MS (43.24), MS+SGA (48.65), SGA (8.11)
Nahas 2003	Academia	2 w	DSM-IV (I or II [39.13%])	HAMD28>1 8	23	HAMD28: 32.66±6.11	60.87	42.90± 8.17	L-HF-rTMS	Sham[§]	Figure-of-eight coil	NR	L-DLPFC	5 cm rule	5	110	10	10	MS (60.87)
Tamas 2007	Academia	4 w	NR (I [100%])**	NR	5	NR	60.00	44.20	R-LF-rTMS	Sham[‡]	Circular coil	Cadwell	R-DLPFC	NR	1	95	NR	8	LAM (20.00), LIT (40.00), QUE (20.00), RIS (20.00), VAL (20.00)
Tavares 2017	Industry	4 w	DSM-IV (I or II [50.00%])	HAMD17>1 7	50	HAMD17: 25.56±4.53	70.00	42.35± 10.52	L-dTMS	Sham coil	H1-coil	Brainswa y dTMS	L-DLPFC	6 cm rule	18	120	1980	20	BE (36.00), LAM (30.00), LIT (60.00), QUE (36.00), VAL (20.00),
Zengin 2022	Academia	2 w	DSM-5 (I or II [20.69%])	HAMD>17	29	HAMD: 20.24±2.65	51.72	40.59 ±9.90	L-HF-rTMS	Sham[§]	Figure-of-eight coil	Neuro MS/D	L-DLPFC	5 cm rule	10	110	1000	20	ARI (51.72), ARI-LAI (3.45), BUP (34.48), CAR (6.90), FLU-LAI (3.45), LAM (58.62), LIT (34.48), MOD (6.90), OLA (27.59), PAL-LAI (10.34), QUE (65.52), RIS (20.69), RIS-LAI (3.45), SSRI (24.14), VAL (58.62), VEN (10.34), ZUC-LAI (6.90),

*The study was included in the systematic review only because they did not report the detailed method of rTMS.

**The study did not determine whether the participants had pharmacological treatment-resistant bipolar depression.

† The lowest score among participants

‡ No detailed information about a sham.

§ Not use a sham coil.

† † The study was included in the network meta-analysis only.

AD: antidepressant(s), AP: antipsychotic(s), ARI: aripiprazole, BD: bipolar disorder, BDI: Beck Depression Inventory, BE: benzodiazepine(s), BL: baseline, B-rTMS: bilateral repetitive transcranial magnetic stimulation, BUP: bupropion, CAR: carbamazepine, CYA: cyamemazine, DS: depressive symptoms, DSM(-TR): Diagnostic and Statistical Manual of Mental Disorders (-Text Revision), EEG: electroencephalography, FLU: flupenthixol, HAMD: Hamilton Depression Rating Scale, HYD: hydroxyzine, Hz: hertz, ICD: International Statistical Classification of Diseases and Related Health Problems, LAI: long acting injection, LAM: lamotrigine, L-DLPFC: left-dorsolateral prefrontal cortex, L-dTMS: left-deep transcranial magnetic stimulation, L-HF-rTMS: left-high frequency-repetitive transcranial magnetic stimulation, LIT: lithium, L-iTBS: left-intermittent theta burst stimulation, MADRS: Montgomery Åsberg Depression Rating Scale, MOD: modafinil, MRI: magnetic resonance imaging, MS: mood stabilizer(s), MT: motor threshold, n: number of individuals, NR= not report, OLA: olanzapine, PAL: paliperidone, QUE: quetiapine, R-cTBS: right-continuous intermittent theta burst stimulation, R-DLPFC: right-dorsolateral prefrontal cortex, RIS: risperidone, R-LF-rTMS: right-low frequency-repetitive transcranial magnetic stimulation, rTMS: repetitive transcranial magnetic stimulation, SD: standard deviation, SGA: second generation antipsychotic(s), SSRI: selective serotonin reuptake inhibitor(s), TRI: tricyclic(s), VAL: valproate, VEN: venlafaxine, w: weeks, ZUC: zuclopenthixol

Table S2.1. PRISMA for Pairwise Meta-Analyses Checklist.

Section and Topic	Item #	Checklist item	Location where item is reported
**TITLE**			
Title	1	Identify the report as a systematic review.	P1
**ABSTRACT**			
Abstract	2	See the PRISMA 2020 for Abstracts checklist.	none
**INTRODUCTION**			
Rationale	3	Describe the rationale for the review in the context of existing knowledge.	P3
Objectives	4	Provide an explicit statement of the objective(s) or question(s) the review addresses.	P3
**METHODS**			
Eligibility criteria	5	Specify the inclusion and exclusion criteria for the review and how studies were grouped for the syntheses.	P4
Information sources	6	Specify all databases, registers, websites, organisations, reference lists and other sources searched or consulted to identify studies. Specify the date when each source was last searched or consulted.	P4
Search strategy	7	Present the full search strategies for all databases, registers and websites, including any filters and limits used.	P4
Selection process	8	Specify the methods used to decide whether a study met the inclusion criteria of the review, including how many reviewers screened each record and each report retrieved, whether they worked independently, and if applicable, details of automation tools used in the process.	P4
Data collection process	9	Specify the methods used to collect data from reports, including how many reviewers collected data from each report, whether they worked independently, any processes for obtaining or confirming data from study investigators, and if applicable, details of automation tools used in the process.	P4
Data items	10a	List and define all outcomes for which data were sought. Specify whether all results that were compatible with each outcome domain in each study were sought (e.g. for all measures, time points, analyses), and if not, the methods used to decide which results to collect.	P4
	10b	List and define all other variables for which data were sought (e.g. participant and intervention characteristics, funding sources). Describe any assumptions made about any missing or unclear information.	P4
Study risk of bias assessment	11	Specify the methods used to assess risk of bias in the included studies, including details of the tool(s) used, how many reviewers assessed each study and whether they worked independently, and if applicable, details of automation tools used in the process.	P4
Effect measures	12	Specify for each outcome the effect measure(s) (e.g. risk ratio, mean difference) used in the synthesis or presentation of results.	P4
Synthesis methods	13a	Describe the processes used to decide which studies were eligible for each synthesis (e.g. tabulating the study intervention characteristics and comparing against the planned groups for each synthesis (item #5)).	P4
	13b	Describe any methods required to prepare the data for presentation or synthesis, such as handling of missing summary statistics, or data conversions.	P4
	13c	Describe any methods used to tabulate or visually display results of individual studies and syntheses.	P4
	13d	Describe any methods used to synthesize results and provide a rationale for the choice(s). If meta-analysis was performed, describe the model(s), method(s) to identify the presence and extent of statistical heterogeneity, and software package(s) used.	P4
	13e	Describe any methods used to explore possible causes of heterogeneity among study results (e.g. subgroup analysis, meta-regression).	P4
	13f	Describe any sensitivity analyses conducted to assess robustness of the synthesized results.	P4
Reporting bias assessment	14	Describe any methods used to assess risk of bias due to missing results in a synthesis (arising from reporting biases).	P4

Section and Topic	Item #	Checklist item	Location where item is reported
Certainty assessment	15	Describe any methods used to assess certainty (or confidence) in the body of evidence for an outcome.	P4
**RESULTS**			
Study selection	16a	Describe the results of the search and selection process, from the number of records identified in the search to the number of studies included in the review, ideally using a flow diagram.	P5
	16b	Cite studies that might appear to meet the inclusion criteria, but which were excluded, and explain why they were excluded.	P5
Study characteristics	17	Cite each included study and present its characteristics.	P5
Risk of bias in studies	18	Present assessments of risk of bias for each included study.	P5
Results of individual studies	19	For all outcomes, present, for each study: (a) summary statistics for each group (where appropriate) and (b) an effect estimate and its precision (e.g. confidence/credible interval), ideally using structured tables or plots.	P5
Results of syntheses	20a	For each synthesis, briefly summarise the characteristics and risk of bias among contributing studies.	P5
	20b	Present results of all statistical syntheses conducted. If meta-analysis was done, present for each the summary estimate and its precision (e.g. confidence/credible interval) and measures of statistical heterogeneity. If comparing groups, describe the direction of the effect.	P5
	20c	Present results of all investigations of possible causes of heterogeneity among study results.	P5
	20d	Present results of all sensitivity analyses conducted to assess the robustness of the synthesized results.	P5
Reporting biases	21	Present assessments of risk of bias due to missing results (arising from reporting biases) for each synthesis assessed.	P5
Certainty of evidence	22	Present assessments of certainty (or confidence) in the body of evidence for each outcome assessed.	P5
**DISCUSSION**			
Discussion	23a	Provide a general interpretation of the results in the context of other evidence.	P5
	23b	Discuss any limitations of the evidence included in the review.	P5
	23c	Discuss any limitations of the review processes used.	P5
	23d	Discuss implications of the results for practice, policy, and future research.	P5
**OTHER INFORMATION**			
Registration and protocol	24a	Provide registration information for the review, including register name and registration number, or state that the review was not registered.	P4
	24b	Indicate where the review protocol can be accessed, or state that a protocol was not prepared.	P4
	24c	Describe and explain any amendments to information provided at registration or in the protocol.	P4
Support	25	Describe sources of financial or non-financial support for the review, and the role of the funders or sponsors in the review.	P6
Competing interests	26	Declare any competing interests of review authors.	P7
Availability of data, code and other materials	27	Report which of the following are publicly available and where they can be found: template data collection forms; data extracted from included studies; data used for all analyses; analytic code; any other materials used in the review.	P8

From: Page MJ, McKenzie JE, Bossuyt PM, Boutron I, Hoffmann TC, Mulrow CD, et al. The PRISMA 2020 statement: an updated guideline for reporting systematic reviews. BMJ 2021;372:n71. doi: 10.1136/bmj.n71

For more information, visit: http://www.prisma-statement.org/

This systematic review and pairwise meta-analysis were conducted according to the Preferred Reporting Items for Systematic Reviews and Meta-Analyses (PRISMA) statement (**Table S2.1.**).[1] At least two authors (TK, KS, MH, and YM) simultaneously and independently conducted the literature search, data extraction, and data entry. Furthermore, the authors double-checked all data for accuracy. Any discrepancies between the authors were resolved by discussion with a third author (NI). The study was registered with the Open Science Framework (https://osf.io/t78rv).

Inclusion criteria and literature search strategy

We performed a systematic literature review according to the PICO strategy ("Patients," adults with bipolar depression; "Intervention," rTMS; "Control," sham; "Outcomes,") (see the following section for details). Our study included randomized, sham-controlled trials lasting at least five days. The exclusion criteria were as follows: (1) studies focusing on specific generations (e.g., children/adolescents or older individuals) because the efficacy and safety of psychotropic drugs in children and older individuals differ from those in adults,[2, 3] (2) studies including individuals with a dual diagnosis of bipolar disorder and other disorders such as substance use disorders because these studies might result in heterogeneity.[2, 3] **Figure S1** shows the information regarding the literature search.

Data synthesis and outcome measures

The outcomes were treatment response (primary), improvement in depressive symptoms, remission rate, all-cause discontinuation, and incidence of mania. **Table S3** presents results of the original study and data synthesis of our meta-analysis.

Data extraction

The authors independently extracted data from all the included studies. All analyses were based on the intention-to-treat or modified intention-to-treat principles. When the data required for the meta-analysis were incomplete, we contacted the original study investigators to obtain the unpublished data. We also searched for missing data in published systematic review articles.

Meta-analysis methods

This pairwise meta-analysis used a random-effects model.[4] We calculated the risk ratio (RR) for dichotomous variables or the standardized mean difference (SMD) for continuous variables with 95% confidence intervals (95% CI). We assessed the heterogeneity of the studies using the I^2 statistics, considering that I^2 of ≥50% indicates considerable heterogeneity.[5] When the pairwise meta-analysis showed significant differences in the primary outcome between the treatment groups, the number needed to treat (NNTB) was estimated.

We performed all statistical analyses using the Review Manager software (version 5.4 for Windows; Cochrane Collaboration, http://tech.cochrane.org/Revman). We assessed the methodological quality of the included studies according to the Cochrane risk-of-bias tool for randomized trials (ROB2) (https://www.riskofbias.info/welcome/rob-2-0-tool). Finally, we used funnel plots and Egger's regression tests to detect publication bias. We excluded the Dolberg 2002 study from meta-analysis due to the lack of detailed information on rTMS treatment.

Table S2.2. PRISMA for Network Meta-Analyses Checklist.

Section/Topic	Item #	Checklist Item	Reported on Page #
**TITLE**			
Title	1	Identify the report as a systematic review *incorporating a network meta-analysis (or related form of meta-analysis).*	1
**ABSTRACT**			
Structured summary	2	Provide a structured summary including, as applicable:<br>**Background:** main objectives<br>**Methods:** data sources; study eligibility criteria, participants, and interventions; study appraisal; and *synthesis methods, such as network meta-analysis.*<br>**Results:** number of studies and participants identified; summary estimates with corresponding confidence/credible intervals; *treatment rankings may also be discussed. Authors may choose to summarize pairwise comparisons against a chosen treatment included in their analyses for brevity.*<br>**Discussion/Conclusions:** limitations; conclusions and implications of findings.<br>**Other:** primary source of funding; systematic review registration number with registry name.	3-
**INTRODUCTION**			
Rationale	3	Describe the rationale for the review in the context of what is already known, *including mention of why a network meta-analysis has been conducted.*	5-
Objectives	4	Provide an explicit statement of questions being addressed, with reference to participants, interventions, comparisons, outcomes, and study design (PICOS).	5-
**METHODS**			
Protocol and registration	5	Indicate whether a review protocol exists and if and where it can be accessed (e.g., Web address); and, if available, provide registration information, including registration number.	6-
Eligibility criteria	6	Specify study characteristics (e.g., PICOS, length of follow-up) and report characteristics (e.g., years considered, language, publication status) used as criteria for eligibility, giving rationale. *Clearly describe eligible treatments included in the treatment network, and note whether any have been clustered or merged into the same node (with justification).*	6-
Information sources	7	Describe all information sources (e.g., databases with dates of coverage, contact with study authors to identify additional studies) in the search and date last searched.	6-
Search	8	Present full electronic search strategy for at least one database, including any limits used, such that it could be repeated.	6-
Study selection	9	State the process for selecting studies (i.e., screening, eligibility, included in systematic review, and, if applicable, included in the meta-analysis).	6-
Data collection process	10	Describe method of data extraction from reports (e.g., piloted forms, independently, in duplicate) and any processes for obtaining and confirming data from investigators.	6-
Data items	11	List and define all variables for which data were sought (e.g., PICOS, funding sources) and any assumptions and simplifications made.	6-

**Geometry of the network**	**S1**	Describe methods used to explore the geometry of the treatment network under study and potential biases related to it. This should include how the evidence base has been graphically summarized for presentation, and what characteristics were compiled and used to describe the evidence base to readers.	6-
Risk of bias within individual studies	12	Describe methods used for assessing risk of bias of individual studies (including specification of whether this was done at the study or outcome level), and how this information is to be used in any data synthesis.	6-
Summary measures	13	State the principal summary measures (e.g., risk ratio, difference in means). *Also describe the use of additional summary measures assessed, such as treatment rankings and surface under the cumulative ranking curve (SUCRA)* values, as well as modified approaches used to present summary findings from meta-analyses.*	6-
Planned methods of analysis	14	Describe the methods of handling data and combining results of studies for each network meta-analysis. This should include, but not be limited to:<br>• *Handling of multi-arm trials;*<br>• *Selection of variance structure;*<br>• *Selection of prior distributions in Bayesian analyses; and*<br>• *Assessment of model fit.*	6-
**Assessment of Inconsistency**	**S2**	Describe the statistical methods used to evaluate the agreement of direct and indirect evidence in the treatment network(s) studied. Describe efforts taken to address its presence when found.	6-
Risk of bias across studies	15	Specify any assessment of risk of bias that may affect the cumulative evidence (e.g., publication bias, selective reporting within studies).	6-
Additional analyses	16	Describe methods of additional analyses if done, indicating which were pre-specified. This may include, but not be limited to, the following:<br>• Sensitivity or subgroup analyses;<br>• Meta-regression analyses;<br>• *Alternative formulations of the treatment network; and*<br>• *Use of alternative prior distributions for Bayesian analyses (if applicable).*	6-
**RESULTS†**			
Study selection	17	Give numbers of studies screened, assessed for eligibility, and included in the review, with reasons for exclusions at each stage, ideally with a flow diagram.	10-
**Presentation of network structure**	**S3**	Provide a network graph of the included studies to enable visualization of the geometry of the treatment network.	10-
**Summary of network geometry**	**S4**	Provide a brief overview of characteristics of the treatment network. This may include commentary on the abundance of trials and randomized patients for the different interventions and pairwise comparisons in the network, gaps of evidence in the treatment network, and potential biases reflected by the network structure.	10-
Study characteristics	18	For each study, present characteristics for which data were extracted (e.g., study size, PICOS, follow-up period) and provide the citations.	10-
Risk of bias within studies	19	Present data on risk of bias of each study and, if available, any outcome level assessment.	10-

	Results of individual studies	20	For all outcomes considered (benefits or harms), present, for each study: 1) simple summary data for each intervention group, and 2) effect estimates and confidence intervals. *Modified approaches may be needed to deal with information from larger networks.*	10-
	Synthesis of results	21	Present results of each meta-analysis done, including confidence/credible intervals. *In larger networks, authors may focus on comparisons versus a particular comparator (e.g. placebo or standard care), with full findings presented in an appendix. League tables and forest plots may be considered to summarize pairwise comparisons.* If additional summary measures were explored (such as treatment rankings), these should also be presented.	10-
	**Exploration for inconsistency**	**S5**	Describe results from investigations of inconsistency. This may include such information as measures of model fit to compare consistency and inconsistency models, *P* values from statistical tests, or summary of inconsistency estimates from different parts of the treatment network.	10-
	Risk of bias across studies	22	Present results of any assessment of risk of bias across studies for the evidence base being studied.	10-
	Results of additional analyses	23	Give results of additional analyses, if done (e.g., sensitivity or subgroup analyses, meta-regression analyses, *alternative network geometries studied, alternative choice of prior distributions for Bayesian analyses*, and so forth).	10-
**DISCUSSION**				
	Summary of evidence	24	Summarize the main findings, including the strength of evidence for each main outcome; consider their relevance to key groups (e.g., healthcare providers, users, and policy-makers).	13-
	Limitations	25	Discuss limitations at study and outcome level (e.g., risk of bias), and at review level (e.g., incomplete retrieval of identified research, reporting bias). *Comment on the validity of the assumptions, such as transitivity and consistency. Comment on any concerns regarding network geometry (e.g., avoidance of certain comparisons).*	13-
	Conclusions	26	Provide a general interpretation of the results in the context of other evidence, and implications for future research.	13-
**FUNDING**				16
	Funding	27	Describe sources of funding for the systematic review and other support (e.g., supply of data); role of funders for the systematic review. This should also include information regarding whether funding has been received from manufacturers of treatments in the network and/or whether some of the authors are content experts with professional conflicts of interest that could affect use of treatments in the network.	

The systematic review and meta-analysis were performed according to the Preferred Reporting Items for Systematic Reviews and Network Meta-Analysis (PRISMA) guidelines (**Table S2.2.**).[6] The study was registered with the Open Science Framework (https://osf.io/sjmhw).

Inclusion criteria and literature search strategy

We performed a systematic literature review in accordance with the PICO strategy ("Patients," adults with bipolar depression; "Intervention," rTMS; "Control," conditions included either sham or other rTMS; "Outcomes," see the following section for detail). Our study included only randomized, controlled trials lasting at least five days. The exclusion criteria were the same as our pairwise meta-analysis. **Figure S1** shows the information regarding the literature search.

Data synthesis and outcome measures

These were the same as our pairwise meta-analysis.

Data extraction

These were the same as our pairwise meta-analysis.

Meta-analysis methods

Frequentist network meta-analysis was performed using the random-effects model.[4] The RR for dichotomous variables or the SMD for continuous variables was calculated with 95% CI. Network heterogeneity was assessed using τ^2 statistics. Statistical evaluation of incoherence was performed using the design-by-treatment test (globally)[7] and the Separate Direct from Indirect Evidence (SIDE) test (locally).[8] The treatments for each outcome were ranked using the surface under the curve cumulative ranking probabilities. The methodological quality of the studies was evaluated using the Cochrane ROB2 (https://www.riskofbias.info/welcome/rob-2-0-tool). The transitivity assumption was tested by extracting potential effect modifiers such as sample size, study duration, and mean age and comparing their distribution across comparisons in the network. We determined whether the distribution differences were large enough to threaten the analysis validity by comparing the distribution of these possible effect modifiers across treatments included in the network meta-analysis using the Kruskal–Wallis test (continuous variables), the Pearson chi-squared test or the Fisher exact test (categorical variables) and by assessing their actual impact on the treatment effect through meta-regression analyses.[9, 10] A meta-regression analysis was performed to determine the relationship of potentially confounding factors (e.g., mean age, proportion of females, total number of participants, publication year, risk of bias, sponsorship, study duration, sham, percent motor threshold, locating method, the total number of pulses/session, the total number of sessions during the study, number of pulse during the study) to the magnitude of the effect on the primary outcome. Funnel plots were created to investigate potential publication bias. Finally, to assess the credibility of the findings of each network meta-analysis, the findings were incorporated into the Confidence in Network Meta-Analysis (CINeMA) application, which is an adaptation of the Grading of Recommendations Assessment, Development, and Evaluation approach.[11-13]

1. Page MJ, McKenzie JE, Bossuyt PM, Boutron I, Hoffmann TC, Mulrow CD *et al.* The PRISMA 2020 statement: an updated guideline for reporting systematic reviews. *BMJ* 2021; **372:** n71.
2. McIntyre RS, Berk M, Brietzke E, Goldstein BI, Lopez-Jaramillo C, Kessing LV *et al.* Bipolar disorders. *Lancet* 2020; **396**(10265)**:** 1841-1856.
3. Herrman H, Patel V, Kieling C, Berk M, Buchweitz C, Cuijpers P *et al.* Time for united action on depression: a Lancet-World Psychiatric Association Commission. *Lancet* 2022; **399**(10328)**:** 957-1022.
4. DerSimonian R, Laird N. Meta-analysis in clinical trials. *Controlled clinical trials* 1986; **7**(3)**:** 177-188.

5. Higgins J, Thomas J, Chandler J, Cumpston M, Li T, Page M *et al.* Cochrane Handbook for Systematic Reviews of Interventions version 6.2. *wwwtrainingcochraneorg/handbook* 2021.

6. Hutton B, Salanti G, Caldwell DM, Chaimani A, Schmid CH, Cameron C *et al.* The PRISMA extension statement for reporting of systematic reviews incorporating network meta-analyses of health care interventions: checklist and explanations. *Ann Intern Med* 2015; **162**(11)**:** 777-784.

7. Jackson D, Barrett JK, Rice S, White IR, Higgins JP. A design-by-treatment interaction model for network meta-analysis with random inconsistency effects. *Stat Med* 2014; **33**(21)**:** 3639-3654.

8. Dias S, Welton NJ, Caldwell DM, Ades AE. Checking consistency in mixed treatment comparison meta-analysis. *Stat Med* 2010; **29**(7-8)**:** 932-944.

9. Cipriani A, Higgins JP, Geddes JR, Salanti G. Conceptual and technical challenges in network meta-analysis. *Ann Intern Med* 2013; **159**(2)**:** 130-137.

10. Ostuzzi G, Bertolini F, Tedeschi F, Vita G, Brambilla P, Del Fabro L *et al.* Oral and long-acting antipsychotics for relapse prevention in schizophrenia-spectrum disorders: a network meta-analysis of 92 randomized trials including 22,645 participants. *World Psychiatry* 2022; **21**(2)**:** 295-307.

11. Salanti G, Del Giovane C, Chaimani A, Caldwell DM, Higgins JP. Evaluating the quality of evidence from a network meta-analysis. *PLoS One* 2014; **9**(7)**:** e99682.

12. Nikolakopoulou A, Higgins JPT, Papakonstantinou T, Chaimani A, Del Giovane C, Egger M *et al.* CINeMA: An approach for assessing confidence in the results of a network meta-analysis. *PLoS Med* 2020; **17**(4)**:** e1003082.

13. Papakonstantinou T, Nikolakopoulou A, Higgins JPT, Egger M, Salanti G. CINeMA: Software for semiautomated assessment of the confidence in the results of network meta-analysis. *Campbell Systematic Reviews* 2020; **16:** e1080.

Table S3. Results of the original study and data synthesis of our meta-analysis

Study	Depression scale	Results of the study	The definition of response	Results of the study	The definition of remission	Results of the study
Beynel 2014	MADRS	L-iTBS = Sham	≥ 50% improvement in MADRS	L-iTBS = Sham	MADRS < 8	L-iTBS = Sham
Bulteau 2019	MADRS	L-iTBS = Sham	≥ 50% improvement in MADRS	L-iTBS = Sham	MADRS < 8	L-iTBS = Sham
Dolberg 2002	HAMD	rTMS > Sham				
Fitzgerald 2016	HAMD17	B-rTMS = Sham	NR	B-rTMS = Sham	NR	B-rTMS = Sham
Hu 2016	HAMD17	L-HF-rTMS = Sham<br>R-LF-rTMS = Sham	> 50% improvement in HAMD17	L-HF-rTMS = Sham<br>R-LF-rTMS = Sham	HAMD17 < 8	L-HF-rTMS = Sham<br>R-LF-rTMS = Sham
Kazemi 2016	BDI	B-rTMS = R-LF-rTMS	≥ 50% improvement in BDI	B-rTMS > R-LF-rTMS	BDI < 8	B-rTMS = R-LF-rTMS
Mak 2021	MADRS	R-LF-rTMS = Sham	≥ 50% improvement in MADRS	R-LF-rTMS = Sham	MADRS < 7	R-LF-rTMS = Sham
Mallik 2022	HAMD17	R-cTBS = Sham				
McGirr 2021	MADRS	L-iTBS = Sham	≥ 50% improvement in MADRS	L-iTBS = Sham	MADRS < 13	L-iTBS = Sham
Nahas 2003	HAMD28	L-HF-rTMS = Sham	> 50% improvement in HAMD28	L-HF-rTMS = Sham	NR	L-HF-rTMS = Sham
Tamas 2007	HAMD21	R-LF-rTMS > Sham				
Tavares 2017	HAMD17	L-dTMS > Sham	≥ 50% improvement in HAMD17	L-dTMS = Sham	HAMD17 < 8	L-dTMS = Sham
Zengin 2022	HAMD	L-HF-rTMS = Sham	≥ 50% improvement in HAMD	L-HF-rTMS > Sham		

[a]A=B: A was similar to B, A > B: A was superior to B.

BDI: Beck Depression Inventory, B-rTMS: bilateral repetitive transcranial magnetic stimulation, HAMD: Hamilton Depression Rating Scale, L-dTMS: left-deep transcranial magnetic stimulation, L-HF-rTMS: left-high frequency-repetitive transcranial magnetic stimulation, L-iTBS: left-intermittent theta burst stimulation, MADRS: Montgomery Åsberg Depression Rating Scale, NR: not report, R-cTBS: right-continuous theta burst stimulation, R-LF-rTMS: right-low frequency-repetitive transcranial magnetic stimulation, rTMS: repetitive transcranial magnetic stimulation

Table S4. Transitivity assessment.

	Boxplot	Kruskal–Wallis equality of populations rank test for continuous variables or the Pearson chi-squared test for binary and categorical variables (or the Fisher exact test whether more than 20% of cells had an expected frequency below 5)
Mean age (K = 13)	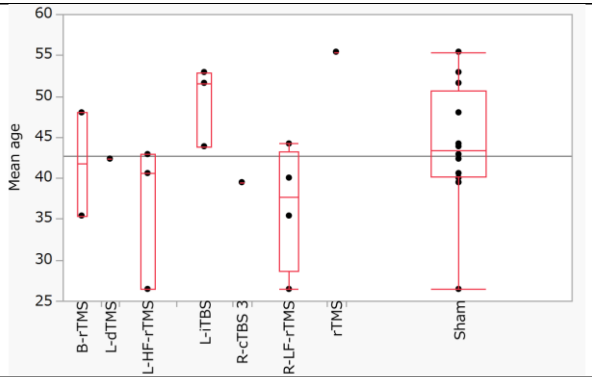	Chi-squared with ties = 9.49 (df = 7), p = 0.22
Proportion of females (K = 13)	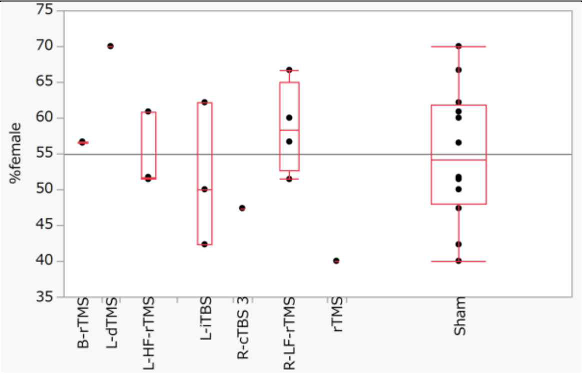	Chi-squared with ties = 7.33 (df = 7), p = 0.40
Total number of participants (K = 13)	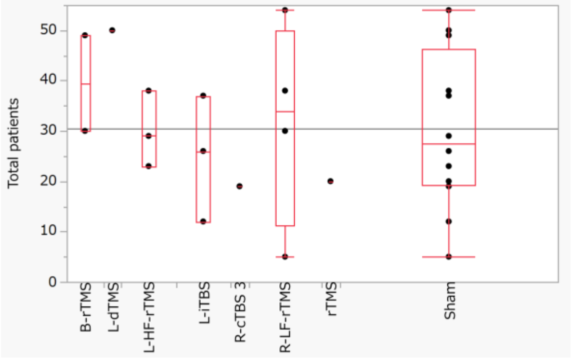	Chi-squared with ties = 5.12 (df = 7), p = 0.65

<table>
<tr><td>Publication year (K = 13)</td><td>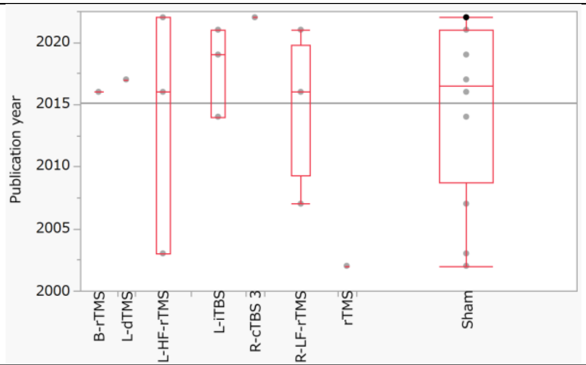
</td><td>Chi-squared with ties = 5.21 (df = 7), p = 0.63</td></tr>
<tr><td>Risk of bias (K= 13)</td><td>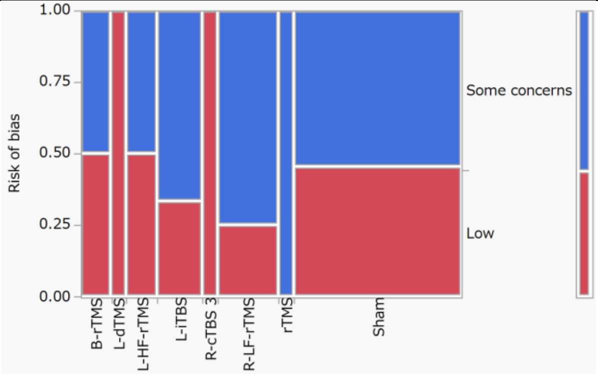
</td><td>Fisher chi^2 = 5.28 (df = 7), p = 0.62</td></tr>
<tr><td>Sponsorship (K = 13)</td><td>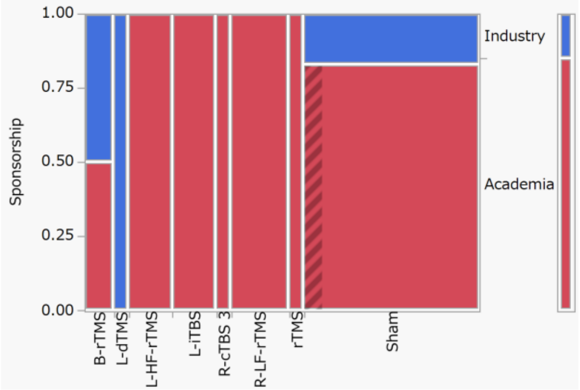
</td><td>Fisher chi^2 = 9.07 (df = 7), p = 0.25</td></tr>
</table>

Study duration (K = 13)	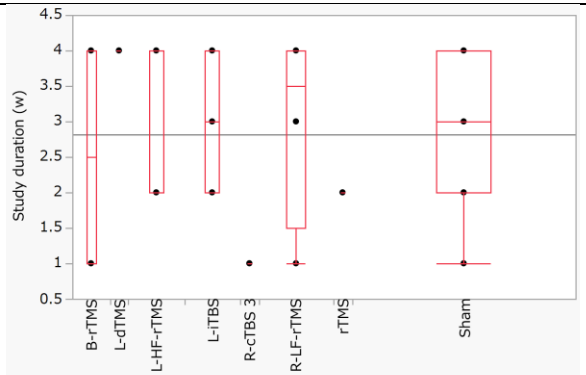	Chi-squared with ties = 4.40 (df = 7), p = 0.73
Sham (K = 10)	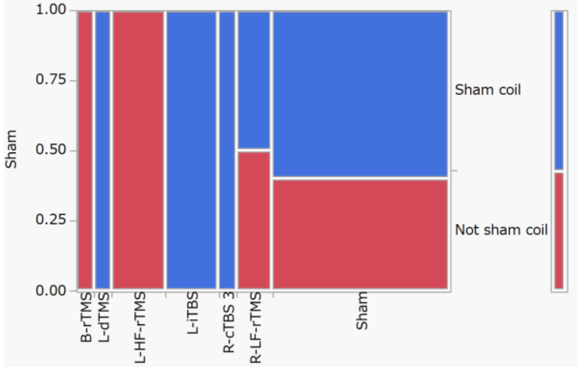	Fisher chi^2 = 12.45 (df = 6), p = 0.18
Percent motor threshold (K = 12)	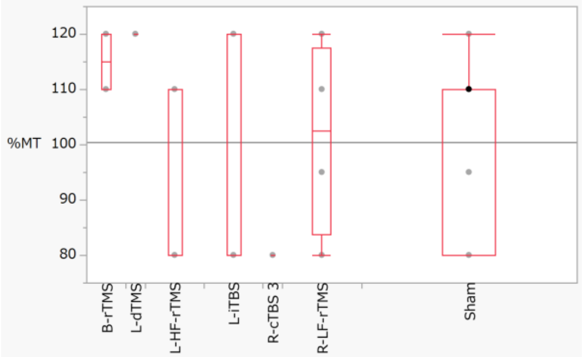	Chi-squared with ties = 5.02 (df = 6), p = 0.54

Locating method (K = 11)	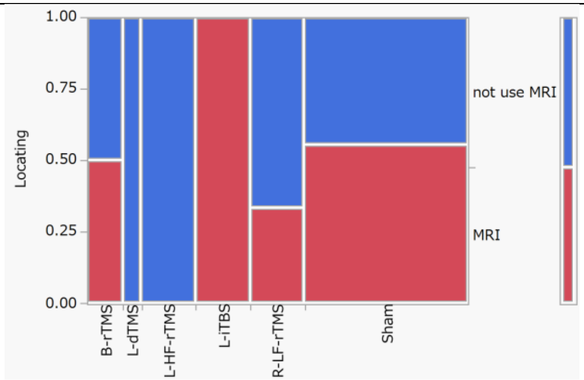	Fisher $chi^2 = 11.04$ (df = 6), p = 0.09
Total number of pulses/session (K = 11)	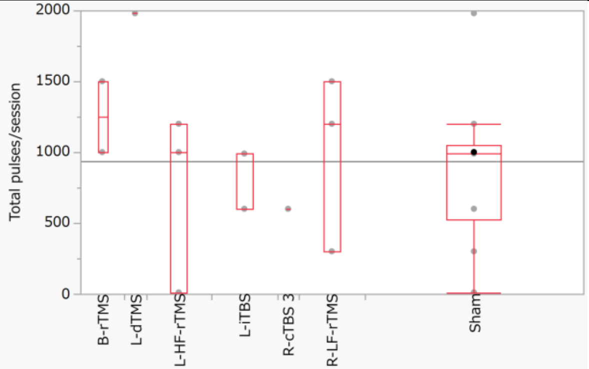	Chi-squared with ties = 5.58 (df = 6), p = 0.47
Total number of sessions during the study (K = 13)	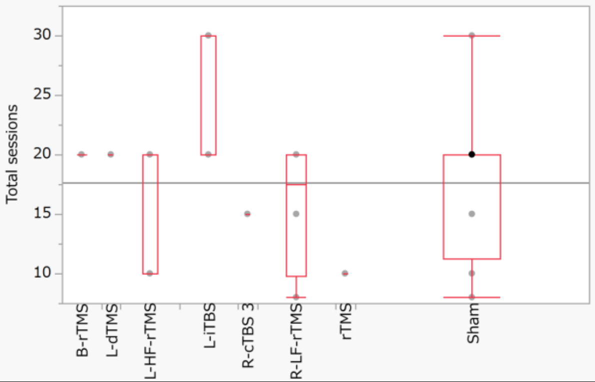	Chi-squared with ties = 4.87 (df = 6), p = 0.56

Total number of pulses during the study (K = 13)	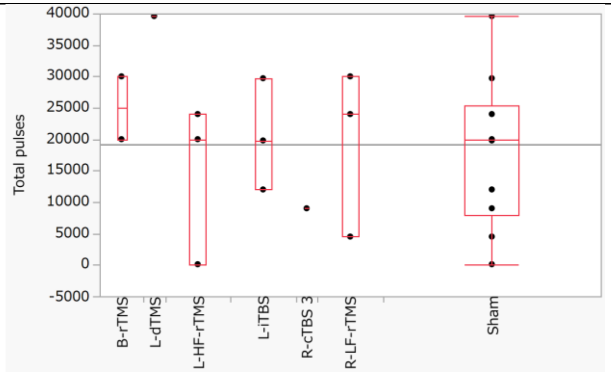	Chi-squared with ties = 6.51 (df = 7), p = 0.48

Appendix S1. Treatment response

Network meta-analysis

10 studies, 342 participants

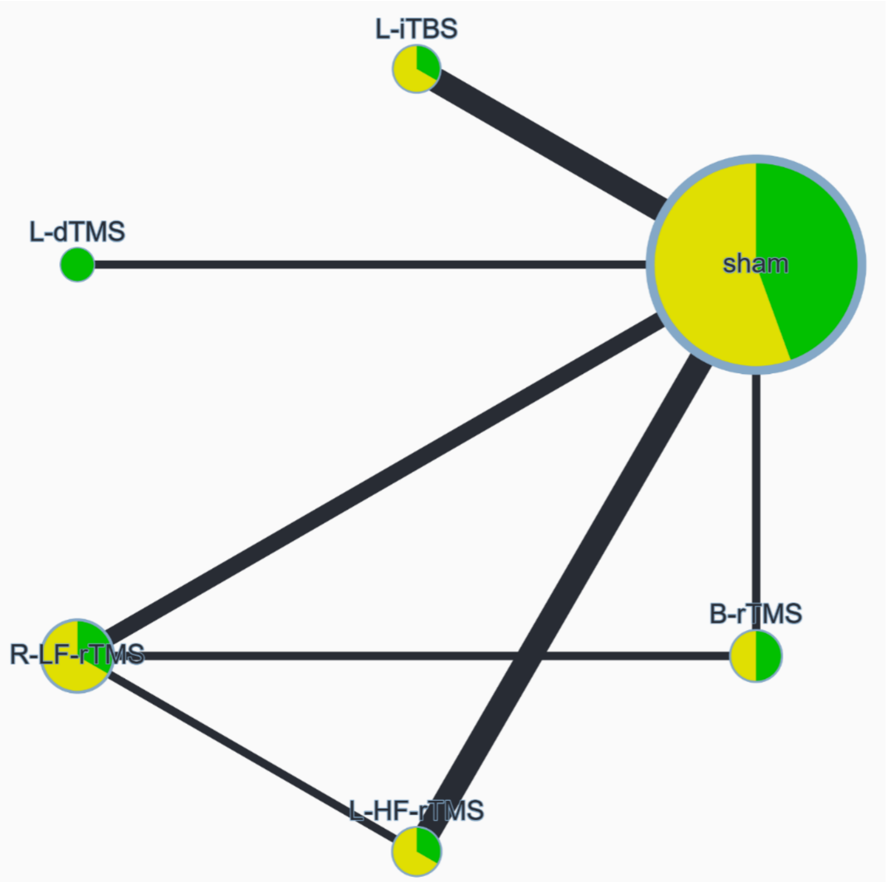

Node size by sample size

Node color by risk of bias

Green: low overall risk of bias

Yellow: moderate overall risk of bias

Edge width by number of studies

League table (risk ratio with 95% confidence interval)

B-rTMS	1.040 (0.353, 3.067)	1.767 (0.851, 3.669)	1.371 (0.576, 3.264)	1.762 (0.989, 3.137)	**2.080 (1.014, 4.268)**
	L-dTMS	1.699 (0.669, 4.315)	1.318 (0.514, 3.384)	1.694 (0.668, 4.296)	2.000 (0.891, 4.488)
		L-HF-rTMS	0.776 (0.396, 1.519)	0.997 (0.627, 1.586)	1.177 (0.740, 1.873)
			L-iTBS	1.285 (0.657, 2.511)	1.517 (0.933, 2.466)
				R-LF-rTMS	1.181 (0.744, 1.873)
					Sham

Global heterogeneity

Global heterogeneity was assessed by means of τ^2 (low: $\tau^2 \leq 0.010$; moderate: $0.010 < \tau^2 \leq 0.242$; high: $\tau^2 > 0.242$).

Huhn M, et al. Lancet 2019;394(10202):939-51

Rhodes KM, et al. J Clin Epidemiol 2015;68(1):52-60

Between study variance (τ^2): 0.000 (heterogeneity assessment: low)

Random-effects design-by-treatment interaction model

χ 2 statistic: 0.424 (3 degrees of freedom), P value: 0.935

Incoherence: SIDE test

	P value
B-rTMS vs R-LF-rTMS	0.728
B-rTMS vs. sham	0.728
L-HF-rTMS vs. R-LF-rTMS	0.713
L-HF-rTMS vs. sham	0.924
R-LF-rTMS vs. sham	0.517

Funnel plot (only sham-controlled trials)

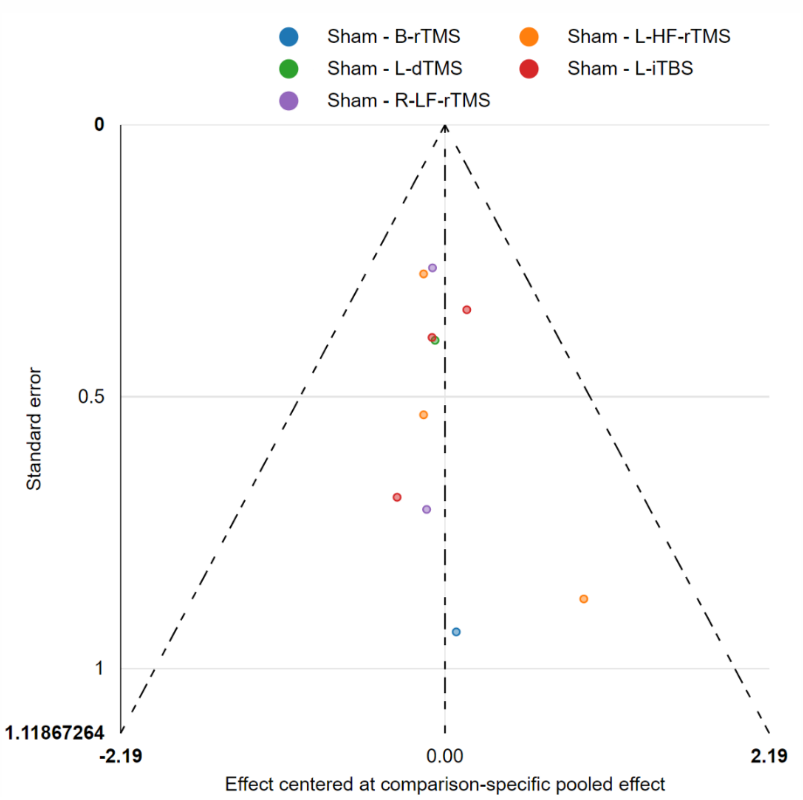

Meta-regression analysis (the sham was the control)

Potential moderators	τ^2	β (SE)
Mean age (K = 10)	0.000	0.283 (0.505)
Proportion of females (K = 10)	0.000	-0.246 (0.554)
Total number of participants (K = 10)	0.000	-0.052 (0.599)
Publication year (K = 10)	0.000	0.226 (0.478)
Risk of bias (K = 10)*	0.000	0.221 (0.479)
Sponsorship (K = 10) †	0.000	-0.124 (3.78)
Duration of study (K = 10)	0.000	-0.210 (0.534)
Sham (K = 9) ‡	0.000	0.099 (0.795)
Percent motor threshold (K = 10)	0.000	0.057 (0.469)
Locating method (K = 10) §	0.000	-0.003 (0.720)
Total number of pulse/session (K = 10)	0.000	0.099 (0.605)
Total number of sessions during the study (K = 10)	0.000	0.246 (0.505)
Total number of pulses during the study (K = 10)	0.000	0.202 (0.559)

*Low overall risk of bias vs. some concerns risk of bias

† Academia vs. industry

‡ Using sham coil vs. not use sham coil

§ Using MRI vs. not using MRI

CINeMA confidence rating

CINeMA is a web application that simplifies the evaluation of confidence in the findings from a network meta-analysis. CINeMA is based on a methodological framework described in the following articles, which consider the following six domains: within-study bias, reporting bias, indirectness, imprecision, heterogeneity, and incoherence. CINeMA grades the confidence in the results of each treatment comparison as high, moderate, low, or very low. If the comparison had only indirect evidence, the comparison was downgraded one level.

Nikolakopoulou A, et al., PLOS Medicine 2020 17 1-19, Papakonstantinou T, et al., Campbell Systematic Reviews 2020 16 e1080

(1) Within-study bias: Risk of bias in RCTs for the main outcomes was assessed independently using the Cochrane risk-of-bias tool for randomized trials (RoB 2).

(2) Reporting bias: Comparison-adjusted funnel plots with less than 10 studies are not meaningful. Therefore, all comparisons were "Suspected."

(3) Indirectness: No indirectness was assumed. Selected rule: Average

(4) Imprecision: For sham comparisons the clinically meaningful threshold was set at a risk ratio of higher or lower than 1. For comparisons of two rTMSs the clinically meaningful threshold was set at risk ratio of 0.8 and 1.25.

(5) Heterogeneity: We used recommendations automatically provided by CINeMA.

(6) Incoherence: We used recommendations automatically provided by CINeMA.

Comparison	Number of studies	Within-study bias	Reporting bias	Indirectness	Imprecision	Heterogeneity	Incoherence	Confidence rating
B-rTMS vs. R-LF-rTMS	1	Some concerns	Some concerns	No concerns	Major concerns	No concerns	No concerns	Low
B-rTMS vs. Sham	1	No concerns	Some concerns	No concerns	No concerns	Major concerns	No concerns	Low
L-HF-rTMS vs. R-LF-rTMS	1	No concerns	Some concerns	No concerns	Major concerns	No concerns	No concerns	Low
L-HF-rTMS vs. Sham	3	No concerns	Some concerns	No concerns	Major concerns	No concerns	No concerns	Low
L-dTMS vs. Sham	1	No concerns	Some concerns	No concerns	Major concerns	No concerns	No concerns	Low
L-iTBS vs. Sham	3	Some concerns	Some concerns	No concerns	Major concerns	No concerns	No concerns	Low
R-LF-rTMS vs. Sham	2	No concerns	Some concerns	No concerns	Major concerns	No concerns	No concerns	Low
B-rTMS vs. L-HF-rTMS	0	No concerns	Some concerns	No concerns	Major concerns	No concerns	No concerns	Very low
B-rTMS vs. L-dTMS	0	No concerns	Some concerns	No concerns	Major concerns	No concerns	No concerns	Very low
B-rTMS vs. L-iTBS	0	Some concerns	Some concerns	No concerns	Major concerns	No concerns	No concerns	Very low
L-dTMS vs. L-HF-rTMS	0	No concerns	Some concerns	No concerns	Major concerns	No concerns	No concerns	Very low
L-HF-rTMS vs. L-iTBS	0	Some concerns	Some concerns	No concerns	Major concerns	No concerns	No concerns	Very low
L-dTMS vs. L-iTBS	0	No concerns	Some concerns	No concerns	Major concerns	No concerns	No concerns	Very low
L-dTMS vs. R-LF-rTMS	0	No concerns	Some concerns	No concerns	Major concerns	No concerns	No concerns	Very low
L-iTBS vs. R-LF-rTMS	0	No concerns	Some concerns	No concerns	Major concerns	No concerns	No concerns	Very low

Appendix S2. Improvement of depressive symptoms

Network meta-analysis

9 studies, 289 participants

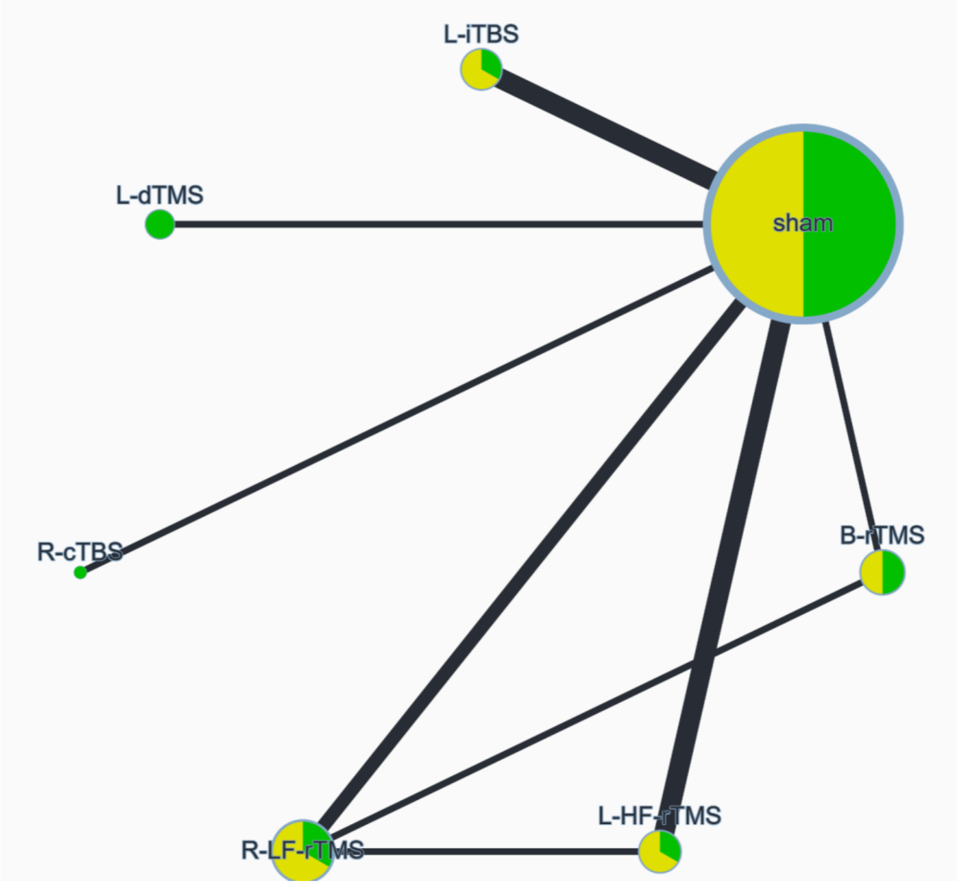

League table (standardized mean difference with 95% confidence interval)

B-rTMS	0.264 (-0.474, 1.003)	0.075 (-0.558, 0.709)	-0.222 (-0.882, 0.437)	-0.321 (-1.323, 0.681)	-0.079 (-0.590, 0.433)	-0.246 (-0.722, 0.231)
	L-dTMS	-0.189 (-0.907, 0.528)	-0.487 (-1.212, 0.238)	-0.586 (-1.632, 0.460)	-0.343 (-1.031, 0.344)	-0.510 (-1.074, 0.054)
		L-HF-rTMS	-0.298 (-0.933, 0.338)	-0.397 (-1.383, 0.590)	-0.154 (-0.695, 0.387)	-0.321 (-0.764, 0.123)
			L-iTBS	-0.099 (-1.091, 0.893)	0.144 (-0.457, 0.745)	-0.023 (-0.478, 0.432)
				R-cTBS	0.242 (-0.722, 1.207)	0.076 (-0.805, 0.957)
					R-LF-rTMS	-0.167 (-0.559, 0.226)
						Sham

Global heterogeneity

Between study variance (τ^2): 0.000 (heterogeneity assessment: low)

Random-effects design-by-treatment interaction model

χ 2 statistic: 1.731 (3 degrees of freedom), P value: 0.630

Incoherence: SIDE test

	P value
B-rTMS vs R-LF-rTMS	0.212
B-rTMS vs. sham	0.212
L-HF-rTMS vs. R-LF-rTMS	0.962
L-HF-rTMS vs. sham	0.984
R-LF-rTMS vs. sham	0.241

CINeMA confidence rating

Comparison	Number of studies	Within-study bias	Reporting bias	Indirectness	Imprecision	Heterogeneity	Incoherence	Confidence rating
B-rTMS vs. R-LF-rTMS	1	Some concerns	Some concerns	No concerns	Major concerns	No concerns	No concerns	Low
B-rTMS vs. sham	1	No concerns	Some concerns	No concerns	Major concerns	No concerns	No concerns	Low
L-HF-rTMS vs. R-LF-rTMS	1	No concerns	Some concerns	No concerns	Major concerns	No concerns	No concerns	Low
L-HF-rTMS vs. sham	3	Some concerns	Some concerns	No concerns	Major concerns	No concerns	No concerns	Low
L-dTMS vs. sham	1	No concerns	Some concerns	No concerns	Major concerns	No concerns	No concerns	Low
L-iTBS vs. sham	3	Some concerns	Some concerns	No concerns	Major concerns	No concerns	No concerns	Low
R-LF-rTMS vs. sham	2	Some concerns	Some concerns	No concerns	Major concerns	No concerns	No concerns	Low
R-cTBS vs. sham	1	No concerns	Some concerns	No concerns	Major concerns	No concerns	No concerns	Low
B-rTMS vs. L-HF-rTMS	0	No concerns	Some concerns	No concerns	Major concerns	No concerns	No concerns	Very low
B-rTMS vs. L-dTMS	0	No concerns	Some concerns	No concerns	Major concerns	No concerns	No concerns	Very low
B-rTMS vs. L-iTBS	0	No concerns	Some concerns	No concerns	Major concerns	No concerns	No concerns	Very low
B-rTMS vs. R-cTBS	0	No concerns	Some concerns	No concerns	Major concerns	No concerns	No concerns	Very low
L-dTMS vs. L-HF-rTMS	0	No concerns	Some concerns	No concerns	Major concerns	No concerns	No concerns	Very low
L-HF-rTMS vs. L-iTBS	0	Some concerns	Some concerns	No concerns	Major concerns	No concerns	No concerns	Very low
L-HF-rTMS vs. R-cTBS	0	No concerns	Some concerns	No concerns	Major concerns	No concerns	No concerns	Very low
L-dTMS vs. L-iTBS	0	No concerns	Some concerns	No concerns	Major concerns	No concerns	No concerns	Very low
L-dTMS vs. R-LF-rTMS	0	No concerns	Some concerns	No concerns	Major concerns	No concerns	No concerns	Very low
L-dTMS vs. R-cTBS	0	No concerns	Some concerns	No concerns	Major concerns	No concerns	No concerns	Very low
L-iTBS vs. R-LF-rTMS	0	Some concerns	Some concerns	No concerns	Major concerns	No concerns	No concerns	Very low
L-iTBS vs. R-cTBS	0	No concerns	Some concerns	No concerns	Major concerns	No concerns	No concerns	Very low
R-cTBS vs. R-LF-rTMS	0	No concerns	Some concerns	No concerns	Major concerns	No concerns	No concerns	Very low

Appendix S3. Remission rate

Pairwise meta-analysis

Study or Subgroup	Weight	Risk Ratio M-H, Random, 95% CI
**1.3.1 B-rTMS**		
Fitzgerald 2016 B-rTMS	3.3%	5.00 [0.25, 98.75]
**Subtotal (95% CI)**	**3.3%**	**5.00 [0.25, 98.75]**
Total events		
Heterogeneity: Not applicable		
Test for overall effect: Z = 1.06 (P = 0.29)		
**1.3.2 L-dTMS**		
Tavares 2017 L-dTMS	24.2%	1.75 [0.58, 5.24]
**Subtotal (95% CI)**	**24.2%**	**1.75 [0.58, 5.24]**
Total events		
Heterogeneity: Not applicable		
Test for overall effect: Z = 1.00 (P = 0.32)		
**1.3.3 L-HF-rTMS**		
Hu 2016 L-HF-rTMS	11.5%	1.64 [0.33, 8.03]
Nahas 2003 L-HF-rTMS	4.1%	1.09 [0.08, 15.41]
**Subtotal (95% CI)**	**15.6%**	**1.47 [0.38, 5.75]**
Total events		
Heterogeneity: Tau² = 0.00; Chi² = 0.07, df = 1 (P = 0.80); I² = 0%		
Test for overall effect: Z = 0.55 (P = 0.58)		
**1.3.4 L-iTBS**		
Beynel 2014 L-iTBS	3.6%	0.27 [0.02, 4.59]
Bulteau 2019 L-iTBS	25.6%	1.46 [0.50, 4.23]
McGirr 2021 L-iTBS	13.5%	1.06 [0.24, 4.57]
**Subtotal (95% CI)**	**42.7%**	**1.14 [0.50, 2.60]**
Total events		
Heterogeneity: Tau² = 0.00; Chi² = 1.27, df = 2 (P = 0.53); I² = 0%		
Test for overall effect: Z = 0.31 (P = 0.75)		
**1.3.5 R-LF-rTMS**		
Hu 2016 R-LF-rTMS	11.3%	1.50 [0.30, 7.43]
Mak 2021 R-LF-rTMS	2.9%	3.22 [0.14, 75.75]
**Subtotal (95% CI)**	**14.3%**	**1.75 [0.42, 7.31]**
Total events		
Heterogeneity: Tau² = 0.00; Chi² = 0.18, df = 1 (P = 0.67); I² = 0%		
Test for overall effect: Z = 0.77 (P = 0.44)		
**Total (95% CI)**	**100.0%**	**1.47 [0.86, 2.52]**
Total events		
Heterogeneity: Tau² = 0.00; Chi² = 2.63, df = 8 (P = 0.96); I² = 0%		
Test for overall effect: Z = 1.40 (P = 0.16)		
Test for subgroup differences: Chi² = 1.16, df = 4 (P = 0.88), I² = 0%		

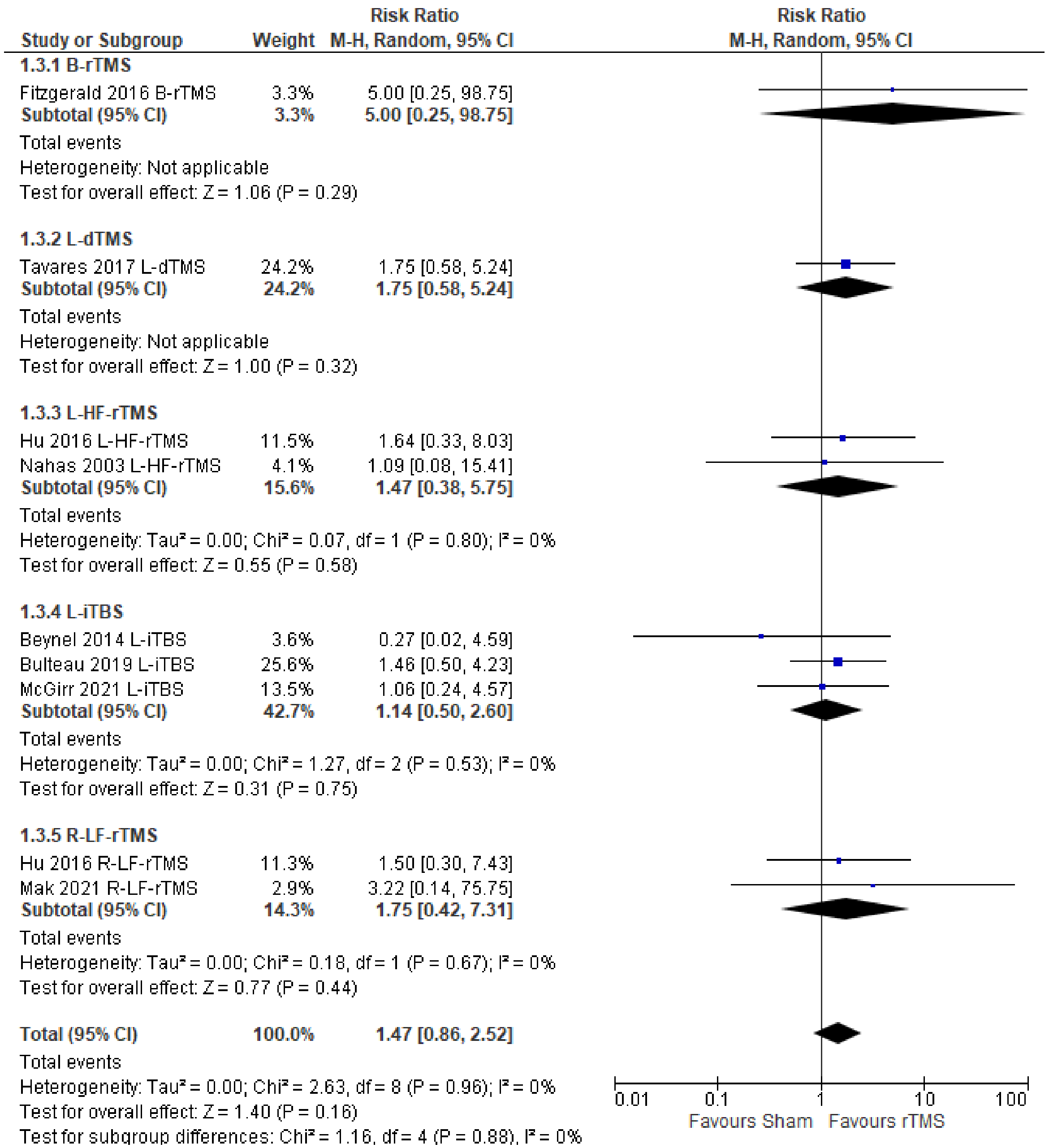

Network meta-analysis

9 studies, 313 participants

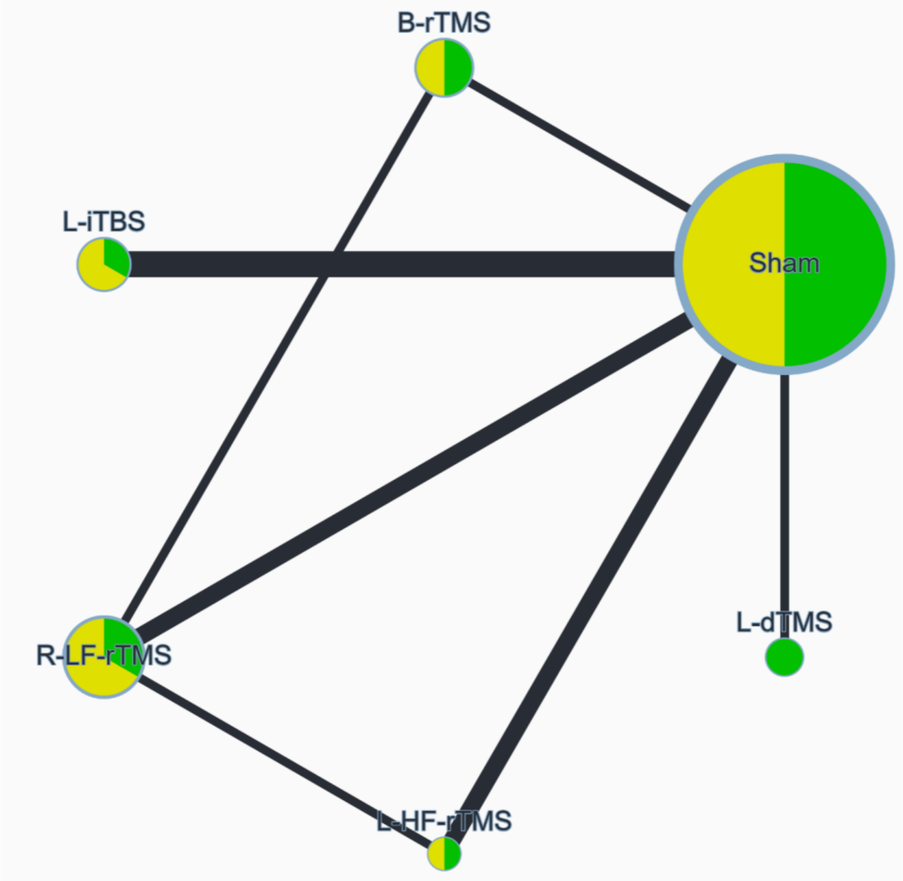

League table (risk ratio with 95% confidence interval)

B-rTMS	1.208 (0.200, 7.291)	1.210 (0.272, 5.376)	1.851 (0.357, 9.599)	1.077 (0.462, 2.512)	2.113 (0.508, 8.788)
	L-dTMS	1.002 (0.184, 5.453)	1.532 (0.389, 6.039)	0.892 (0.169, 4.703)	1.750 (0.585, 5.238)
		L-HF-rTMS	1.529 (0.330, 7.076)	0.890 (0.249, 3.188)	1.746 (0.480, 6.354)
			L-iTBS	0.582 (0.130, 2.601)	1.142 (0.501, 2.604)
				R-LF-rTMS	1.962 (0.562, 6.848)
					Sham

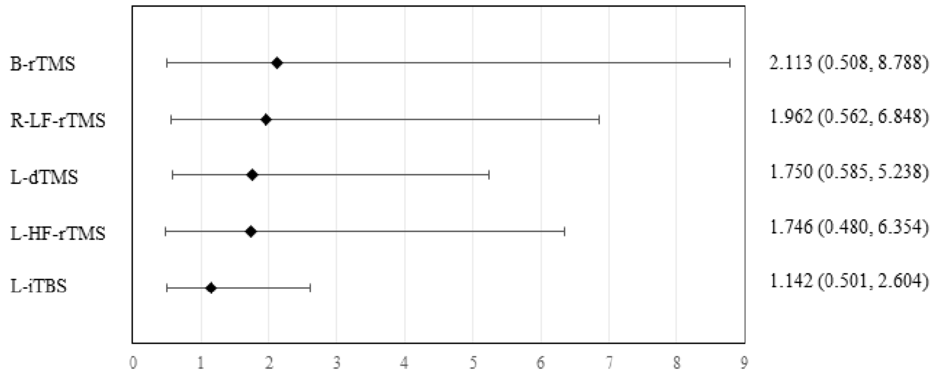

Global heterogeneity

Between study variance (τ^2): 0.000 (heterogeneity assessment: low)

Random-effects design-by-treatment interaction model

χ 2 statistic: 0.699 (3 degrees of freedom), P value: 0.873

Incoherence: SIDE test

	P value
B-rTMS vs R-LF-rTMS	0.519
B-rTMS vs. sham	0.519
L-HF-rTMS vs. R-LF-rTMS	0.438
L-HF-rTMS vs. sham	0.440
R-LF-rTMS vs. sham	0.750

CINeMA confidence rating

Comparison	Number of studies	Within-study bias	Reporting bias	Indirectness	Imprecision	Heterogeneity	Incoherence	Confidence rating
B-rTMS vs. R-LF-rTMS	1	Some concerns	Some concerns	No concerns	Major concerns	No concerns	No concerns	Low
B-rTMS vs. Sham	1	No concerns	Some concerns	No concerns	Major concerns	No concerns	No concerns	Low
L-HF-rTMS vs. R-LF-rTMS	1	No concerns	Some concerns	No concerns	Major concerns	No concerns	No concerns	Low
L-HF-rTMS vs. Sham	2	No concerns	Some concerns	No concerns	Major concerns	No concerns	No concerns	Low
L-dTMS vs. Sham	1	No concerns	Some concerns	No concerns	Major concerns	No concerns	No concerns	Low
L-iTBS vs. Sham	3	Some concerns	Some concerns	No concerns	Major concerns	No concerns	No concerns	Low
R-LF-rTMS vs. Sham	2	No concerns	Some concerns	No concerns	Major concerns	No concerns	No concerns	Low
B-rTMS vs. L-HF-rTMS	0	No concerns	Some concerns	No concerns	Major concerns	No concerns	No concerns	Very low
B-rTMS vs. L-dTMS	0	No concerns	Some concerns	No concerns	Major concerns	No concerns	No concerns	Very low
B-rTMS vs. L-iTBS	0	Some concerns	Some concerns	No concerns	Major concerns	No concerns	No concerns	Very low
L-dTMS vs. L-HF-rTMS	0	No concerns	Some concerns	No concerns	Major concerns	No concerns	No concerns	Very low
L-HF-rTMS vs. L-iTBS	0	No concerns	Some concerns	No concerns	Major concerns	No concerns	No concerns	Very low
L-dTMS vs. L-iTBS	0	No concerns	Some concerns	No concerns	Major concerns	No concerns	No concerns	Very low
L-dTMS vs. R-LF-rTMS	0	No concerns	Some concerns	No concerns	Major concerns	No concerns	No concerns	Very low
L-iTBS vs. R-LF-rTMS	0	No concerns	Some concerns	No concerns	Major concerns	No concerns	No concerns	Very low

Appendix S4. All-cause discontinuation

Pairwise meta-analysis

Study or Subgroup	Weight	Risk Ratio M-H, Random, 95% CI
**1.4.1 B-rTMS**		
Fitzgerald 2016 B-rTMS	16.0%	2.00 [0.41, 9.87]
**Subtotal (95% CI)**	**16.0%**	**2.00 [0.41, 9.87]**
Total events		
Heterogeneity: Not applicable		
Test for overall effect: Z = 0.85 (P = 0.39)		
**1.4.2 L-dTMS**		
Tavares 2017 L-dTMS	17.1%	2.50 [0.53, 11.70]
**Subtotal (95% CI)**	**17.1%**	**2.50 [0.53, 11.70]**
Total events		
Heterogeneity: Not applicable		
Test for overall effect: Z = 1.16 (P = 0.24)		
**1.4.3 L-HF-rTMS**		
Hu 2016 L-HF-rTMS	5.8%	1.08 [0.08, 15.46]
Nahas 2003 L-HF-rTMS		Not estimable
Zengin 2022 L-HF-rTMS	15.2%	1.61 [0.31, 8.24]
**Subtotal (95% CI)**	**21.0%**	**1.44 [0.36, 5.80]**
Total events		
Heterogeneity: Tau² = 0.00; Chi² = 0.06, df = 1 (P = 0.80); I² = 0%		
Test for overall effect: Z = 0.52 (P = 0.61)		
**1.4.4 L-iTBS**		
McGirr 2021 L-iTBS	16.5%	0.53 [0.11, 2.54]
**Subtotal (95% CI)**	**16.5%**	**0.53 [0.11, 2.54]**
Total events		
Heterogeneity: Not applicable		
Test for overall effect: Z = 0.80 (P = 0.43)		
**1.4.5 R-cTBS**		
Mallik 2022 R-cTBS	5.9%	0.73 [0.05, 9.97]
**Subtotal (95% CI)**	**5.9%**	**0.73 [0.05, 9.97]**
Total events		
Heterogeneity: Not applicable		
Test for overall effect: Z = 0.24 (P = 0.81)		
**1.4.6 R-LF-rTMS**		
Hu 2016 R-LF-rTMS	5.7%	1.00 [0.07, 14.34]
Mak 2021 R-LF-rTMS	17.9%	1.08 [0.24, 4.87]
**Subtotal (95% CI)**	**23.6%**	**1.06 [0.28, 3.93]**
Total events		
Heterogeneity: Tau² = 0.00; Chi² = 0.00, df = 1 (P = 0.96); I² = 0%		
Test for overall effect: Z = 0.08 (P = 0.93)		
**Total (95% CI)**	**100.0%**	**1.26 [0.67, 2.39]**
Total events		
Heterogeneity: Tau² = 0.00; Chi² = 2.60, df = 7 (P = 0.92); I² = 0%		
Test for overall effect: Z = 0.72 (P = 0.47)		
Test for subgroup differences: Chi² = 2.53, df = 5 (P = 0.77), I² = 0%		

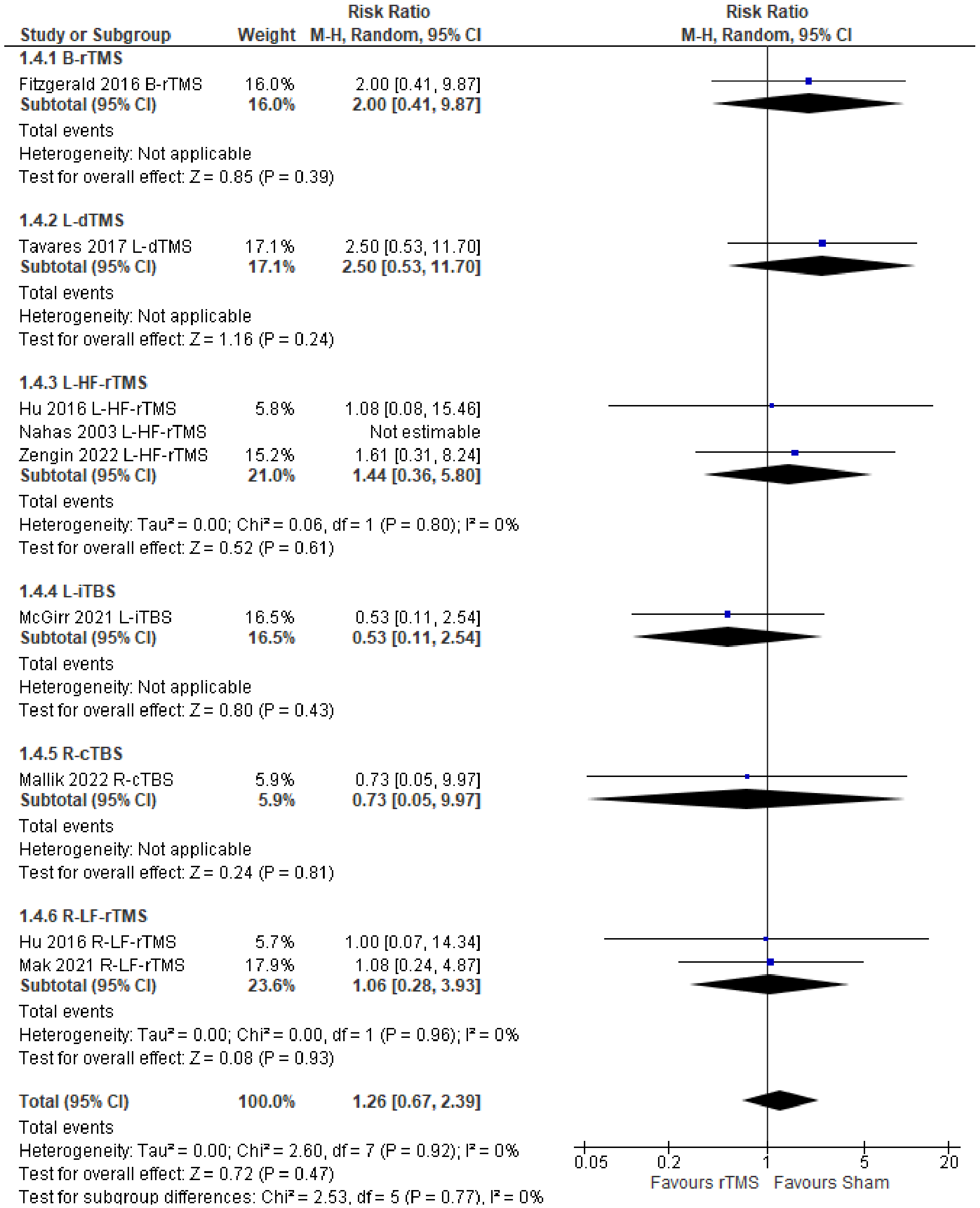

Network meta-analysis

9 studies, 326 participants

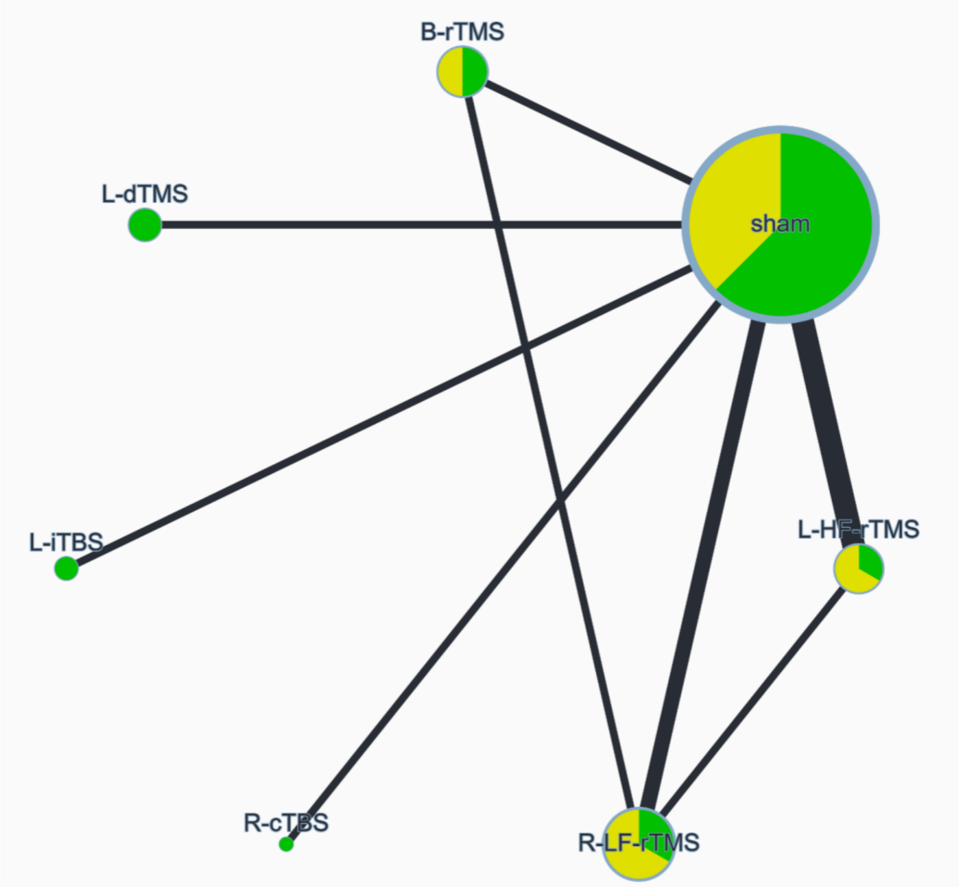

League table (risk ratio with 95% confidence interval)

B-rTMS	0.738 (0.087, 6.285)	1.319 (0.190, 9.168)	3.496 (0.403, 30.353)	2.537 (0.125, 51.460)	1.602 (0.263, 9.751)	1.845 (0.418, 8.148)
	L-dTMS	1.788 (0.242, 13.206)	4.737 (0.524, 42.820)	3.437 (0.165, 71.787)	2.172 (0.303, 15.538)	2.500 (0.534, 11.700)
		L-HF-rTMS	2.650 (0.351, 19.989)	1.923 (0.105, 35.318)	1.215 (0.238, 6.210)	1.398 (0.392, 4.989)
			L-iTBS	0.726 (0.034, 15.365)	0.458 (0.063, 3.350)	0.528 (0.110, 2.537)
				R-cTBS	0.632 (0.035, 11.351)	0.727 (0.053, 9.969)
					R-LF-rTMS	1.151 (0.340, 3.903)
						Sham

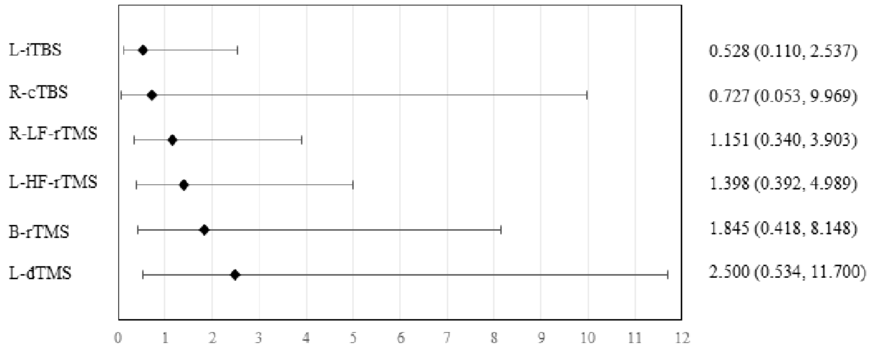

Global heterogeneity

Between study variance (τ^2): 0.000 (heterogeneity assessment: low)

Random-effects design-by-treatment interaction model

χ 2 statistic: 0.121 (3 degrees of freedom), P value: 0.989

Incoherence: SIDE test

	P value
B-rTMS vs R-LF-rTMS	0.786
B-rTMS vs. sham	0.786
L-HF-rTMS vs. R-LF-rTMS	0.915
L-HF-rTMS vs. sham	0.990
R-LF-rTMS vs. sham	0.731

CINeMA confidence rating

Comparison	Number of studies	Within-study bias	Reporting bias	Indirectness	Imprecision	Heterogeneity	Incoherence	Confidence rating
B-rTMS vs. R-LF-rTMS	1	Some concerns	Some concerns	No concerns	Major concerns	No concerns	No concerns	Low
B-rTMS vs. sham	1	No concerns	Some concerns	No concerns	Major concerns	No concerns	No concerns	Low
L-HF-rTMS vs. R-LF-rTMS	1	Some concerns	Some concerns	No concerns	Major concerns	No concerns	No concerns	Low
L-HF-rTMS vs. sham	3	Some concerns	Some concerns	No concerns	Major concerns	No concerns	No concerns	Low
L-dTMS vs. sham	1	No concerns	Some concerns	No concerns	Major concerns	No concerns	No concerns	Low
L-iTBS vs. sham	1	No concerns	Some concerns	No concerns	Major concerns	No concerns	No concerns	Low
R-LF-rTMS vs. sham	2	Some concerns	Some concerns	No concerns	Major concerns	No concerns	No concerns	Low
R-cTBS vs. sham	1	No concerns	Some concerns	No concerns	Major concerns	No concerns	No concerns	Low
B-rTMS vs. L-HF-rTMS	0	No concerns	Some concerns	No concerns	Major concerns	No concerns	No concerns	Very low
B-rTMS vs. L-dTMS	0	No concerns	Some concerns	No concerns	Major concerns	No concerns	No concerns	Very low
B-rTMS vs. L-iTBS	0	No concerns	Some concerns	No concerns	Major concerns	No concerns	No concerns	Very low
B-rTMS vs. R-cTBS	0	No concerns	Some concerns	No concerns	Major concerns	No concerns	No concerns	Very low
L-dTMS vs. L-HF-rTMS	0	No concerns	Some concerns	No concerns	Major concerns	No concerns	No concerns	Very low
L-HF-rTMS vs. L-iTBS	0	No concerns	Some concerns	No concerns	Major concerns	No concerns	No concerns	Very low
L-HF-rTMS vs. R-cTBS	0	No concerns	Some concerns	No concerns	Major concerns	No concerns	No concerns	Very low
L-dTMS vs. L-iTBS	0	No concerns	Some concerns	No concerns	Major concerns	No concerns	No concerns	Very low
L-dTMS vs. R-LF-rTMS	0	No concerns	Some concerns	No concerns	Major concerns	No concerns	No concerns	Very low
L-dTMS vs. R-cTBS	0	No concerns	Some concerns	No concerns	Major concerns	No concerns	No concerns	Very low
L-iTBS vs. R-LF-rTMS	0	No concerns	Some concerns	No concerns	Major concerns	No concerns	No concerns	Very low
L-iTBS vs. R-cTBS	0	No concerns	Some concerns	No concerns	Major concerns	No concerns	No concerns	Very low
R-cTBS vs. R-LF-rTMS	0	No concerns	Some concerns	No concerns	Major concerns	No concerns	No concerns	Very low

Appendix S5. Incidence of mania

Pairwise meta-analysis

Study or Subgroup	Weight	Risk Ratio M-H, Random, 95% CI
**1.5.1 L-dTMS**		
Tavares 2017 L-dTMS		Not estimable
**Subtotal (95% CI)**		**Not estimable**
Total events		
Heterogeneity: Not applicable		
Test for overall effect: Not applicable		
**1.5.2 L-HF-rTMS**		
Hu 2016 L-HF-rTMS	11.7%	3.25 [0.15, 72.36]
Nahas 2003 L-HF-rTMS		Not estimable
Zengin 2022 L-HF-rTMS		Not estimable
**Subtotal (95% CI)**	**11.7%**	**3.25 [0.15, 72.36]**
Total events		
Heterogeneity: Not applicable		
Test for overall effect: Z = 0.74 (P = 0.46)		
**1.5.3 L-iTBS**		
Bulteau 2019 L-iTBS		Not estimable
McGirr 2021 L-iTBS	11.5%	3.16 [0.14, 72.84]
**Subtotal (95% CI)**	**11.5%**	**3.16 [0.14, 72.84]**
Total events		
Heterogeneity: Not applicable		
Test for overall effect: Z = 0.72 (P = 0.47)		
**1.5.4 R-LF-rTMS**		
Hu 2016 R-LF-rTMS		Not estimable
Mak 2021 R-LF-rTMS	76.8%	2.77 [0.82, 9.31]
Tamas 2007 R-LF-rTMS		Not estimable
**Subtotal (95% CI)**	**76.8%**	**2.77 [0.82, 9.31]**
Total events		
Heterogeneity: Not applicable		
Test for overall effect: Z = 1.65 (P = 0.10)		
**Total (95% CI)**	**100.0%**	**2.86 [0.99, 8.29]**
Total events		
Heterogeneity: Tau² = 0.00; Chi² = 0.01, df = 2 (P = 0.99); I² = 0%		
Test for overall effect: Z = 1.94 (P = 0.05)		
Test for subgroup differences: Chi² = 0.01, df = 2 (P = 0.99), I² = 0%		

Risk Ratio
M-H, Random, 95% CI

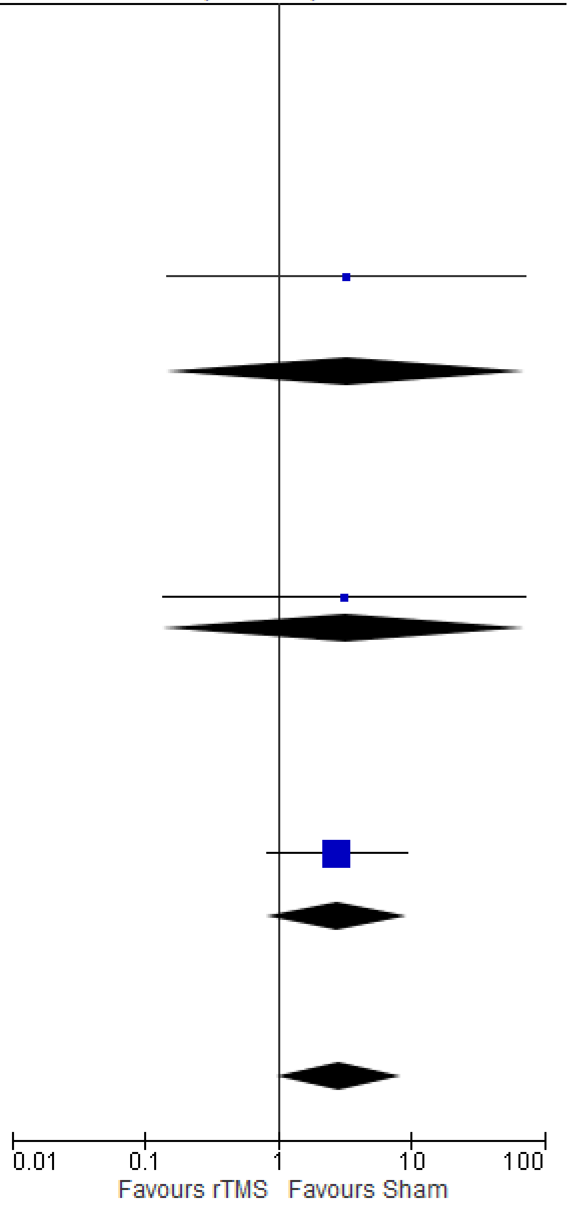

Network meta-analysis

9 studies, 289 participants

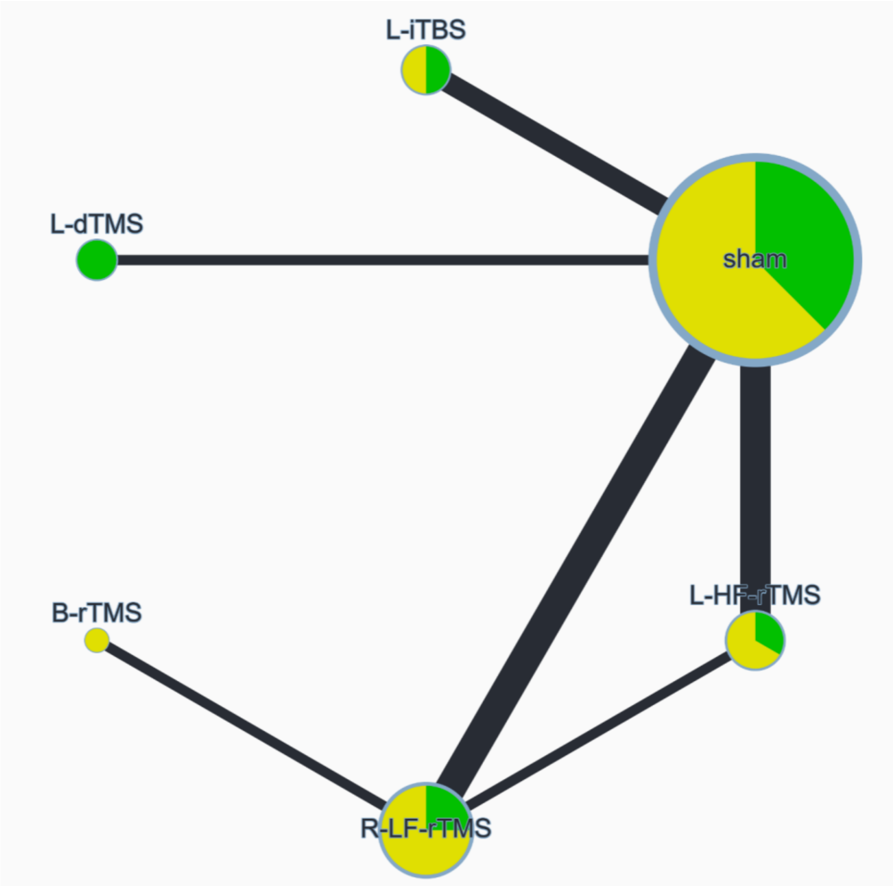

League table (risk ratio with 95% confidence interval)

B-rTMS	1.985 (0.008, 525.158)	0.823 (0.011, 62.659)	0.937 (0.009, 101.596)	1.000 (0.021, 47.285)	1.985 (0.036, 109.049)
	L-dTMS	0.415 (0.006, 30.384)	0.472 (0.005, 46.016)	0.504 (0.009, 28.352)	1.000 (0.021, 48.487)
		L-HF-rTMS	1.139 (0.054, 23.980)	1.215 (0.169, 8.758)	2.412 (0.384, 15.153)
			L-iTBS	1.067 (0.074, 15.295)	2.118 (0.186, 24.085)
				R-LF-rTMS	1.985 (0.670, 5.881)
					Sham

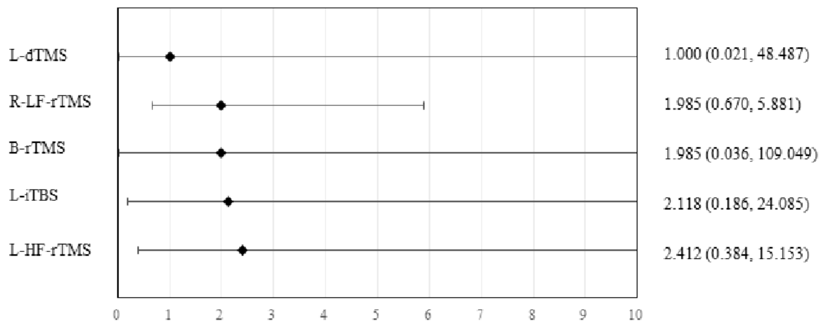

Global heterogeneity

Between study variance (τ^2): 0.000 (heterogeneity assessment: low)

Random-effects design-by-treatment interaction model

χ 2 statistic: 0.780 (2 degrees of freedom), P value: 0.677

Incoherence: SIDE test

	P value
L-HF-rTMS vs. R-LF-rTMS	0.418
L-HF-rTMS vs. sham	0.478
R-LF-rTMS vs. sham	0.431

CINeMA confidence rating

Comparison	Number of studies	Within-study bias	Reporting bias	Indirectness	Imprecision	Heterogeneity	Incoherence	Confidence rating
B-rTMS vs. R-LF-rTMS	1	Some concerns	Some concerns	No concerns	Major concerns	No concerns	No concerns	Low
L-HF-rTMS vs. R-LF-rTMS	1	Some concerns	Some concerns	No concerns	Major concerns	No concerns	No concerns	Low
L-HF-rTMS vs. sham	3	Some concerns	Some concerns	No concerns	Major concerns	No concerns	No concerns	Low
L-dTMS vs. sham	1	No concerns	Some concerns	No concerns	Major concerns	No concerns	No concerns	Low
L-iTBS vs. sham	2	No concerns	Some concerns	No concerns	Major concerns	No concerns	No concerns	Low
R-LF-rTMS vs. sham	3	Some concerns	Some concerns	No concerns	Major concerns	No concerns	No concerns	Low
B-rTMS vs. L-HF-rTMS	0	Some concerns	Some concerns	No concerns	Major concerns	No concerns	No concerns	Very low
B-rTMS vs. L-dTMS	0	Some concerns	Some concerns	No concerns	Major concerns	No concerns	No concerns	Very low
B-rTMS vs. L-iTBS	0	Some concerns	Some concerns	No concerns	Major concerns	No concerns	No concerns	Very low
B-rTMS vs. sham	0	Some concerns	Some concerns	No concerns	Major concerns	No concerns	No concerns	Very low
L-dTMS vs. L-HF-rTMS	0	No concerns	Some concerns	No concerns	Major concerns	No concerns	No concerns	Very low
L-HF-rTMS vs. L-iTBS	0	No concerns	Some concerns	No concerns	Major concerns	No concerns	No concerns	Very low
L-dTMS vs. L-iTBS	0	No concerns	Some concerns	No concerns	Major concerns	No concerns	No concerns	Very low
L-dTMS vs. R-LF-rTMS	0	No concerns	Some concerns	No concerns	Major concerns	No concerns	No concerns	Very low
L-iTBS vs. R-LF-rTMS	0	Some concerns	Some concerns	No concerns	Major concerns	No concerns	No concerns	Very low
